# Active restoration efforts drive community succession and assembly in a desert during the past 53 years

**DOI:** 10.1002/eap.3068

**Published:** 2024-11-25

**Authors:** Qingqing Hou, Weigang Hu, Ying Sun, Elly Morriën, Qiang Yang, Muhammad Aqeel, Qiajun Du, Junlan Xiong, Longwei Dong, Shuran Yao, Jie Peng, Yuan Sun, Muhammad Adnan Akram, Rui Xia, Yahui Zhang, Xiaoting Wang, Shubin Xie, Liang Wang, Liang Zhang, Fan Li, Yan Deng, Jiali Luo, Jingyan Yuan, Quanlin Ma, Karl J. Niklas, Jinzhi Ran, Jianming Deng

**Affiliations:** ^1^ State Key Laboratory of Herbage Improvement and Grassland Agro‐Ecosystems College of Ecology, Lanzhou University Lanzhou China; ^2^ Department of Ecosystem and Landscape Dynamics (IBED‐ELD) Institute for Biodiversity and Ecosystem Dynamics, University of Amsterdam Amsterdam The Netherlands; ^3^ Gansu Academy of Forestry Lanzhou China; ^4^ School of Integrative Plant Science, Cornell University Ithaca New York USA

**Keywords:** community assembly, desertification, ecological restoration, plant‐bacteria‐fungi correlations, stochasticity, succession, temporal thresholds

## Abstract

Regreening efforts in deserts have been implemented globally to combat land degradation and desert expansion, but how they affect above‐ and belowground community succession and assembly processes remains unknown. Here, we examined variations in plant and soil microbial community attributes along a 53‐year restoration chronosequence following the establishment of straw checkerboard barriers (SCBs) in the Tengger Desert of China. This approach is a combination of fixing shifting sand and adding organic material (straw) simultaneously to expedite vegetation restoration by enhancing the success of plant establishment. Our findings revealed that the establishment of SCBs significantly triggered plant and soil microbial communities to gradually approximate those of the natural community along restoration duration. We observed positive and negative bidirectional shifts in plant and soil microbial community composition. Critical temporal threshold zones for relatively rapid changes in community composition were identified, with 2–15.5 years for plants, 0.5–8.5 years for bacteria, and 2–8.5 years for fungi. This suggests a delayed response of plant communities to restoration efforts compared with soil microbial communities. Both stochastic and deterministic processes regulated plant and soil microbial community assembly. Stochastic processes played a more important role in plant and fungal community succession, whereas deterministic processes primarily governed bacterial succession. In terms of deterministic processes, temporal variations in community composition mainly resulted from the intrinsic correlations among plant, bacterial, and fungal communities, as well as an increase in soil organic carbon (SOC) with restoration duration. Thus, temporal patterns and functional contributions of bacterial communities appear to be more predictable than those of plant and fungal communities during desert ecosystem restoration. This study emphasizes that plant‐bacteria‐fungi correlations and increasing SOC content are critical for accelerating community succession and promoting dryland restoration. Future studies should explore and integrate temporal variations and restoration effects of multiple ecosystem functions to better predict dryland development and resilience to global climate changes over a large temporal scale.

## INTRODUCTION

In the vast expanses of drylands, the twin challenges of climate change and anthropogenic activities have escalated aridity, triggering widespread land degradation and desertification (Huang et al., 2016; Newbold et al., 2015; Reynolds et al., 2007). This unfolding crisis jeopardizes over 20% of the terrestrial surface, imperiling essential ecosystem functions and structural attributes. Such ramifications seriously threaten the 2 billion people living in drylands globally (Berdugo et al., 2020). In response to this challenge, large‐scale active restoration efforts are supported by governments worldwide (Benayas et al., 2009; Li et al., 2021; Young et al., 2005). Over the last 70 years, China has adopted 13 major national dryland conservation and restoration programs, notably China's Great Green Wall and regreening the desert programs, significantly enhancing cover in 45.76% of China's drylands (Bryan et al., 2018; Li et al., 2021; Ouyang et al., 2016).

**FIGURE 1:**
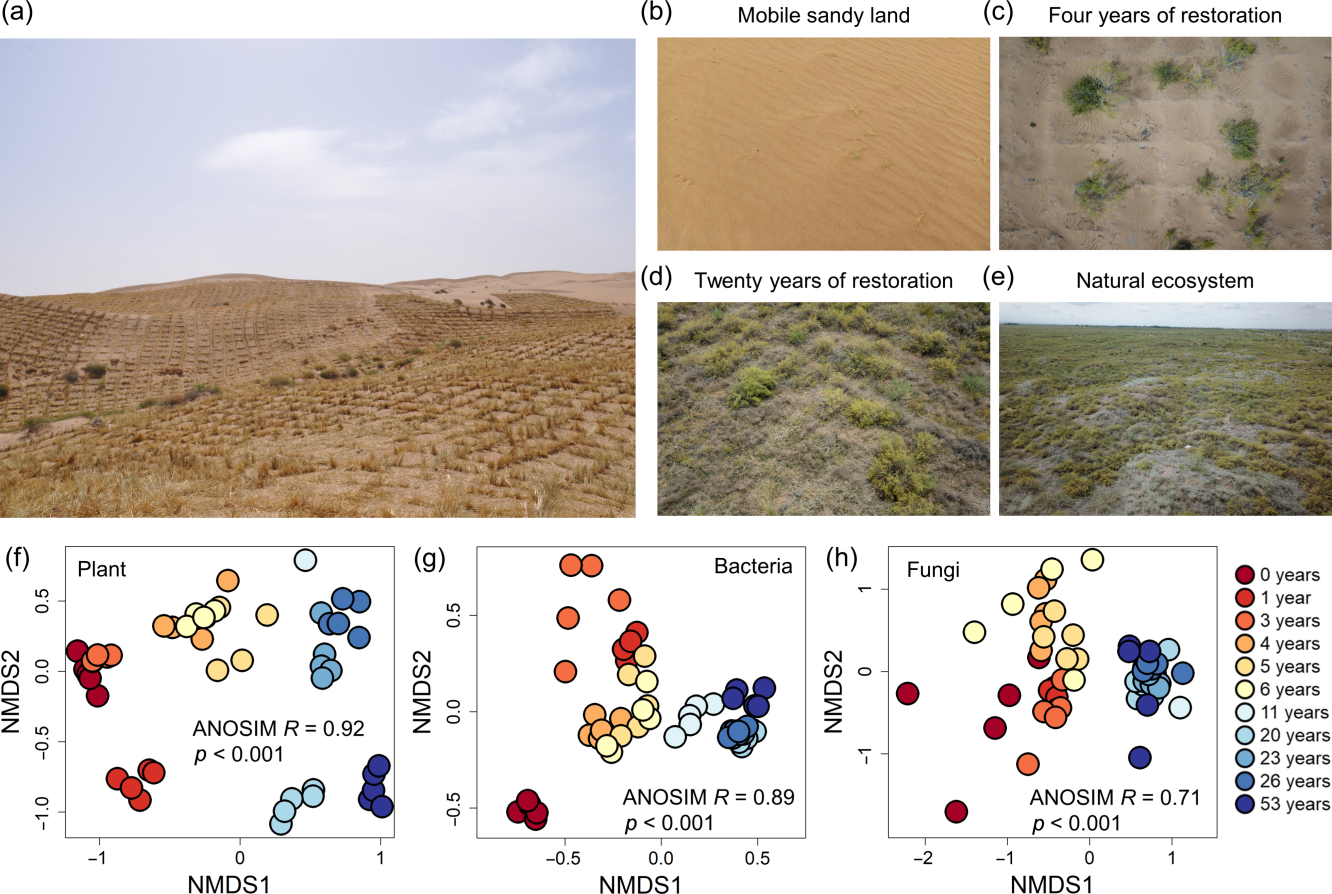
Straw checkerboard barriers (SCBs) established in Northwest China and nonmetric multidimensional scaling (NMDS) ordination of the temporal changes in plant and soil microbial communities based on Bray–Curtis distance. Annual SCBs were established in deserts to prevent the rapid invasion of shifting sands and restore the desertified ecosystems in Northwest China, serving as a typical example (a). Landscapes before and after SCBs were established show a restored trajectory of desert ecosystems over time (b–e). The compositions of the plant (f), soil bacterial (g), and fungal communities (h) are distributed according to the restoration duration gradient. Analysis of similarity (ANOSIM) was performed to test the differences in plant and soil microbial communities among restoration durations. Photograph of SCBs (a) by Quanlin Ma; photographs of landscapes before and after SCBs (b–e) by Qingqing Hou.

An instrumental tool in the restoration arsenal is the straw checkerboard barrier (SCB), a widely endorsed and cost‐effective strategy for revitalizing desert ecosystems. SCBs have been successfully deployed in drylands of northern China, such as the Tengger Desert, Mu Us Desert, and Taklimakan Desert, and their use has also been extended to regions in Africa, East Asia and Mongolia (Li et al., 2006). The barriers are designed in a checkerboard pattern composed of numerous squares (usually 1 × 1 m or 1.2 × 1.2 m), firmly embedded in mobile sandy land (Figure 1a). Compared with other approaches, such as shelterbelts and wind‐break walls, the effectiveness of SCBs in restoring desert is underscored by three key aspects: (1) reducing surface wind speed and mitigate wind erosion, thereby stabilizing shifting sand and creating the necessary prerequisites for biological colonization (Zhang et al., 2018); (2) enhancing dust capture and intercept water and nutrients from surface runoff after rainfall events, thereby leading to greater biological activity compared with adjacent areas (Li et al., 2006; Liu et al., 2013); and (3) creating a source of carbon and nutrients through self‐decomposition, which simultaneously fuels the soil micro‐food‐web, thereby accelerating the reconstruction of above‐ and belowground communities (Li et al., 2007; Luo et al., 2021). However, it remains unclear how the above‐ and belowground communities recover over time following the establishment of SCBs.

Plants and soil microbes are integral components of terrestrial ecosystems, underpinning essential ecosystem functions and processes, such as photosynthesis, organic matter decomposition, and nutrient cycling (Maestre et al., 2012; Zhou et al., 2012). Traditional studies on community succession and ecological restoration have mostly focused on unidirectional variations in simple indicators, such as species richness, community structure, and the relative abundance of dominant taxa, along with their responses to environmental variables (Gao et al., 2019; Holmes & Matlack, 2018). However, this single‐dimensional approach may obscure the multivariate responses of multispecies ecological communities, as well as their temporal patterns, thereby underestimating or misrepresenting the effects of restoration efforts on species distributions and community dynamics (Baker & King, 2010). Therefore, to effectively assess community succession, it is crucial to identify temporal thresholds in ecological communities (i.e., the critical times when community composition and structure undergo relatively rapid changes). This assessment tool for community succession requires integrating the strength and direction of species responses at several time points following restoration (Baker & King, 2010).

In addition to environmental variables, plant‐bacteria‐fungi correlations play important roles in driving both above‐ and belowground community succession (Bauer et al., 2015; Lozano et al., 2014; van der Putten et al., 2013). Prior to plant colonization, soil bacteria and fungi drive soil development and biogeochemical cycling through decomposition and metabolic activities, facilitating early plant community establishment (Nemergut et al., 2007). As soil microbial communities develop, they influence plant growth and community composition both positively and negatively, through symbiosis, pathogenesis, and changes in soil structure and nutrient cycling (Kardol et al., 2006; Reynolds et al., 2003). In turn, plants alter soil bacterial and fungal communities in various ways, such as by providing carbon inputs through root exudates and litter (Bardgett & Walker, 2004). Plant‐bacteria‐fungi correlations may be more influential in driving community succession compared with abiotic factors in localized areas with low environmental heterogeneity, such as the desertification restoration zone in this study. However, these correlations are often overlooked in many restoration studies. Consequently, our understanding of community succession, particularly in terms of temporal thresholds and plant‐bacteria‐fungi correlations, remains inadequate, thereby impeding future efforts to restore natural biogeochemical cycles and nutrient fluxes (Baker & King, 2010; Banerjee et al., 2018; Moreno‐Mateos et al., 2020; Poorter et al., 2021).

Given the ecological significance of understanding the succession and assembly of plant and soil microbial communities, there is a compelling need for a thorough investigation of above‐ and belowground communities over the course of long‐term restoration efforts. To this end, we collected datasets on plant and soil microbial communities from a chronosequence encompassing 11 stages spanning a 53‐year period (1964–2017) of ecological restoration using SCBs established on the southern margin of the Tengger Desert in China. To evaluate the restoration effects, corresponding data were also collected from natural ecosystems. Building upon this background, we formulated the following three hypotheses: (1) the composition and structure of plant and soil microbial communities will gradually resemble those of natural communities over time following the establishment of SCBs, with soil microbial communities responding to restoration efforts earlier than plant communities; (2) biotic variables (plant‐bacteria‐fungi correlations) will play a more important role in driving temporal variations in plant and soil microbial communities during restoration compared with soil environmental variables; and (3) deterministic processes will primarily mediate the assembly of plant and soil microbial communities during ecological restoration.

## MATERIALS AND METHODS

### Field survey and sampling

The research area is situated on the southern margin (37°35′55″ N, 103°50′31″ E) of the Tengger Desert in northwest China, which is seriously impacted by land degradation and desertification due to wind erosion (Li et al., 2021). SCBs were first successfully implemented in this region during the 1950s as a standard method to safeguard the Baotou‐Lanzhou Railway from the effects of shifting sand (Li et al., 2006). Since then, SCBs have proven to be one of the most widespread and economical techniques within the national desert restoration program. In practice, the establishment of SCBs involves arranging wheat straw in a checkerboard pattern composed of numerous squares. Half of the straw is buried in the sand to a depth of approximately 20 cm to prevent displacement, while the other half is exposed to the wind (Zhang et al., 2018). The interior of each straw‐surrounded square is initially bare, as most planted seedlings do not survive beyond one or two years due to harsh environmental conditions and rodent damage. Once established, SCBs are not artificially maintained and rely entirely on the initial conditions and subsequent local environmental conditions. In most cases, SCBs persist for about 5 years until they are completely decomposed by microbes and eroded by wind and surface runoff. To date, hundreds of square kilometers of SCBs have been constructed in the southern Tengger Desert to protect transportation routes, alleviate soil erosion, and restore desertified ecosystems.

In this study, a well‐documented restoration chronosequence spanning 53 years through the establishment of SCBs was identified. Field data were collected between August and September 2017 from 10 plots restored by SCB treatment, each with different restoration durations (i.e., 1, 3, 4, 5, 6, 11, 20, 23, 26, and 53 years). Additionally, data were also collected from a mobile sandy land plot (i.e., an unrestored desertified ecosystem with a restoration duration of 0 years) and three adjacent non‐desertified natural plots (i.e., reference ecosystems). In general, natural plots are situated within close proximity to restored areas (<1 km), due to the nested landscapes of desertified and non‐desertified patches across the study area. The mean elevation, mean annual temperature, and mean annual precipitation are 1680 m above sea level, 8.3°C, and 179 mm, respectively. The aridity index (AI), which is the ratio of precipitation to potential evapotranspiration, has not changed significantly at different stages from 1964 to 2017 (Appendix S1: Figure S1). Plant communities on mobile sandy land are characterized by sparse and small *Agriophyllum squarrosum*, approximately 5 cm in height, with a plant cover of around 2% (Figure 1b). In contrast, the vegetation in non‐desertified natural ecosystems is dominated by *Artemisia ordosica*, standing at a height of 50–100 cm, and featuring a plant cover of approximately 45% (Figure 1e). This type of vegetation is typical of drylands in the region, referred to as *A. ordosica* deserts.

In each plot, a 12 × 12 m quadrat was established in the most representative and homogeneous area. Following the line‐intercept protocol described by Maestre et al. (2012), five 12‐m‐long transects (spaced 1.2 m apart) were arranged within each quadrat to survey the plant community (Appendix S1: Figure S2). Additionally, five 1.2 × 1.2 m subplots, spaced at least 4 m apart, were randomly selected within each quadrat (Appendix S1: Figure S2). In each subplot, 5–7 soil cores (0–10 cm depth) were collected randomly, combined, and homogenized in the field. A total of 70 soil samples were collected from the 14 quadrats. All soil samples were immediately stored in portable car refrigerators and transported to the laboratory. The samples were sieved (<2 mm) and divided into two subsamples. Half of the samples were stored at 4°C for the determination of physical and chemical properties, while the other half were stored at −80°C for DNA extraction (within one week).

### Physicochemical data measurement

Soil organic carbon (SOC) was determined by volumetric methods (ferrous sulfate titration after oxidation with potassium dichromate). Soil ammonium and nitrate nitrogen were measured with a TOC‐TN analyzer (Skalar, Breda, Netherlands). Soil total phosphorus (STP) was determined using a molybdate colorimetric test after perchloric acid digestion. Soil available phosphorus (SAP) was assessed through a 0.5 M NaHCO_3_ extraction (Olsen & Sommers, 1982). Soil moisture was measured gravimetrically after drying samples at 105°C for 48 h. Soil pH was determined using a pH meter in a 1:2.5 mass:volume soil and water suspension.

### Plant phylogeny reconstruction

For the reconstruction of plant phylogeny, sequences of the chloroplast gene rbcL were carefully selected for 29 angiosperms from 14 quadrats (Chen et al., 2016; Lu et al., 2018). All available sequences for the target DNA region were obtained from GenBank using the R package “seqinr” (Roquet et al., 2013). In instances where multiple sequences existed for the same species, preference was given to the longest sequence (Lu et al., 2018). Notably, *Psammochloa villosa* was excluded from the phylogeny due to the unavailability of its target sequence. Consequently, our phylogeny comprised 28 species from 27 genera across 8 families.

### Soil DNA extraction, sequencing, and processing

Soil DNA was extracted from 0.5 g of defrosted soil by using the PowerSoil DNA Isolation Kit (Mo Bio Laboratories, Carlsbad, CA, USA) according to the manufacturer's instructions and stored at −80°C. The universal primer set 338F (5′‐ACTCCTACGGGAGGCAGCAG‐3′) and 806R (5′‐GGACTACHVGGGTWTCTAAT‐3′) was used to amplify the V3−V4 hypervariable regions of the bacterial 16S rRNA gene, and the primer set ITS1F (5′‐CTTGGTCATTTAGAGGAAGTAA‐3′) and ITS2R (5′‐GCTGCGTTCTTCATCGATGC‐3′) targeted the fungal internal transcribed spacer (ITS) region 1 (Hu et al., 2021). The 16S rRNA and ITS amplicons were paired‐end sequenced (2 × 300 bp) on an Illumina MiSeq platform (Illumina, San Diego, CA, USA) at the Majorbio Bio‐pharm Technology Co., Ltd. (Shanghai, China) following standard protocols. The raw sequences were processed and analyzed using the Quantitative Insights into Microbial Ecology (QIIME2 v.2018.6) pipeline (Caporaso et al., 2010). Reads were quality‐trimmed with a threshold of an average quality score >20 over 10 bp moving‐window sizes and a minimum length of 50 bp. Paired‐end reads with at least 10 bp overlap and <5% mismatches were merged into full‐length sequences using the Fast Length Adjustment of Short reads (FLASH) program (Magoč & Salzberg, 2011). Further quality control removed sequences containing ambiguous bases or a Phred score <20 over the entire read length. The UCHIME algorithm checked for chimeric 16S rDNA sequences against the “Gold” database and performed de novo (abundance‐based) chimera detection for ITS sequences (Edgar et al., 2011). Operational taxonomic units (OTUs) were clustered at 97% similarity using UCLUST, and singletons were discarded. The taxonomic identity of representative sequences from each OTU was determined using the Ribosomal Database Project (RDP) Classifier (Wang et al., 2007) against the SILVA database v.128 release (Quast et al., 2013) for bacterial OTUs and the UNITE database v.7.0 release (Kõljalg et al., 2013) for fungal OTUs. To ensure equal sampling depth, 10,000 sequences and 34,139 sequences were randomly selected for bacteria and fungi, respectively, for each sample.

### Statistical analyses

Before statistical analysis, SOC and ammonium nitrogen were log_10_ transformed to improve the normality and homoscedasticity of residuals. Temporal patterns of plant and soil microbial communities were visualized using nonmetric multidimensional scaling (NMDS) ordination based on Bray–Curtis distance. Analysis of similarity (ANOSIM) was used to test the differences in plant and soil microbial communities between restored and natural plots. Mantel test was employed to measure the relationships among the Bray–Curtis dissimilarities of plant, soil bacterial, and fungal communities. To further evaluate the relative importance of biotic (the other two community structures) and abiotic (soil environmental variables) factors on plant and soil bacterial and fungal community structures, we quantified these community structures using the first axis of NMDS analysis based on Bray–Curtis dissimilarity and subsequently conducted a Random Forest analysis using the R package “randomForest.”

Threshold indicator species analysis (TITAN) with the R package “TITAN2” was used to identify negative (*z*−, relative abundance decreased) and positive (*z*+, relative abundance increased) indicator species of plant and soil microbial communities in response to restoration duration and environmental variables and to calculate community‐level thresholds (see Appendix S1: Section S1 for more details; Baker & King, 2010). We constructed a correlation network including soil environmental variables and indicator species of plant and soil microbial communities in response to restoration duration. Differences in connection strengths among environmental variables and indicator species and among indicator species were evaluated using the nonparametric Mann–Whitney *U* test. A similar correlation network including only indicator species was also constructed to assess their modularity and identify keystone species. To quantify the relative abundance of each module for each sample, we first standardized the relative abundance of each node within each module using *z‐*score transformation. The *z‐*scores of all nodes within each module were then averaged to obtain the relative abundance of each module for each sample (Delgado‐Baquerizo et al., 2018). We further evaluated the relationships between the relative abundance of each module and restoration duration, as well as soil environmental variables. Additional details on correlation network analyses are provided in Appendix S1: Section S1.

We inferred the relative contributions of deterministic (i.e., variable selection and homogeneous selection) and stochastic (i.e., dispersal limitation coupled with drift, homogenizing dispersal and drift acting alone) processes to plant and soil microbial community assembly using a null‐modeling‐based quantitative framework based on both phylogenetic and taxonomic turnover between communities (for a detailed description of the method, see Appendix S1: Section S1; Stegen et al., 2012, 2013, 2015). Additionally, we examined relationships between the relative importance of stochasticity in controlling soil microbial community succession and soil environmental variables.

In view of the important role of SOC in soil microbial community assembly and composition, the *z*+ and *z*− indicator OTUs of soil bacterial and fungal communities in response to SOC were identified using TITAN and further grouped into two ecological clusters based on their habitat preferences: (1) high SOC (indicating a preference for high SOC environments) and (2) low SOC (indicating a preference for low SOC environments). We assessed the relationships between SOC, restoration duration, and the relative abundance of these two ecological clusters, considering both linear and nonlinear regressions. Networks were constructed solely on positive correlations (ρ > 0.6 and False Discovery Rate [FDR]‐corrected *p* < 0.001) for bacteria and fungi to elucidate the co‐occurrence patterns of OTUs sharing ecological preferences. To reduce the network complexity, we included all fungal indicator OTUs and those bacterial OTUs with the top 50% indicator scores.

## RESULTS

### Temporal changes in plant and soil microbial community composition

**FIGURE 2:**
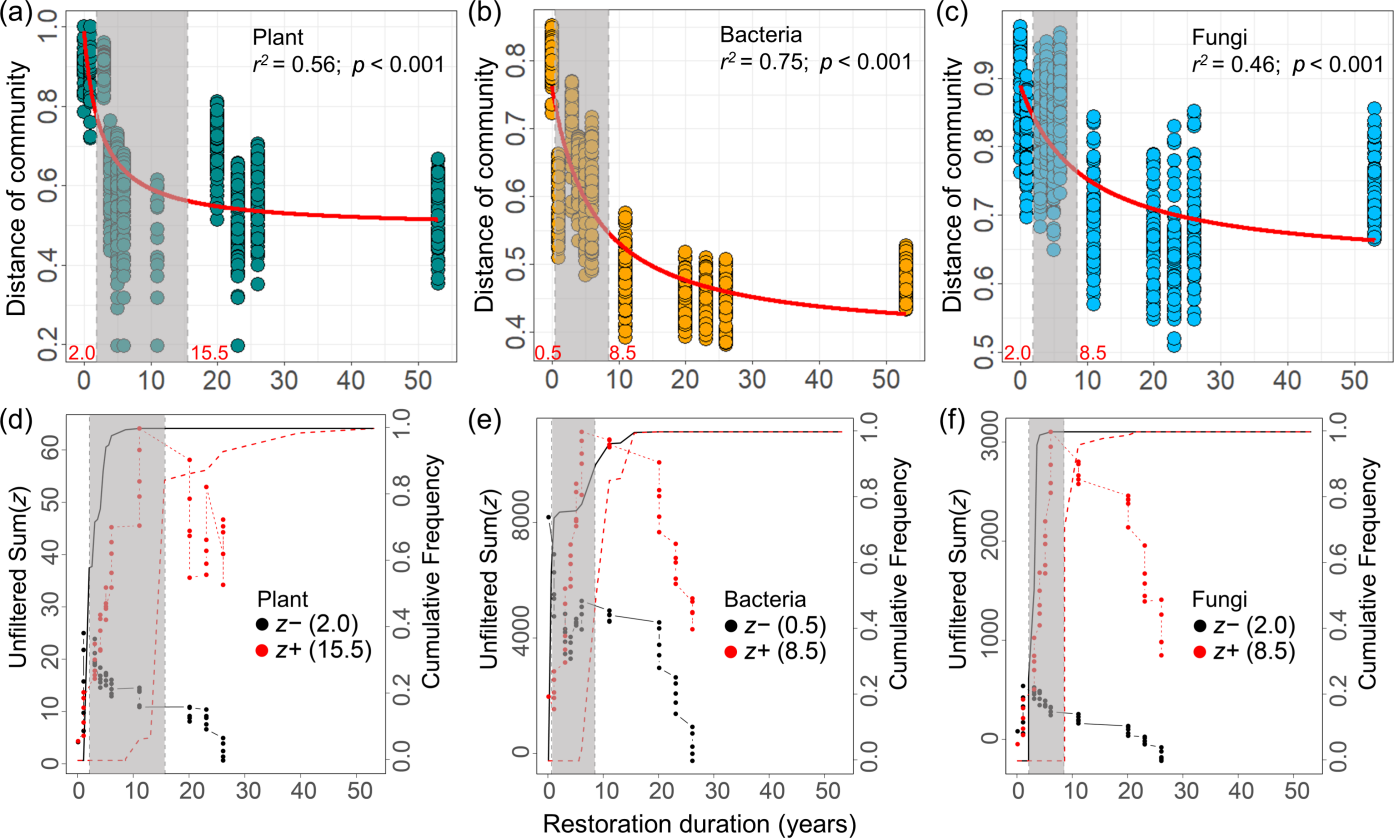
Nonlinear responses of community differences between restored and natural ecosystems and cumulative *z*‐scores at the community level to restoration duration. Nonlinear responses of community differences between restored and natural ecosystems to restoration duration for plants (a), soil bacteria (b), and fungi (c). The red fitted lines are from nonlinear regression. Cumulative positive (sum *z*+) and negative (sum *z*−) responses of plant (d), soil bacterial (e), and fungal (f) communities to restoration duration. Black and red dots represent the sum (*z*−) and sum (*z*+), respectively. Vertical dashed lines indicate temporal thresholds at the community level corresponding to the largest sum (*z*) values. Note that, gray shadings in a–c indicate the temporal threshold zones for plant and soil microbial communities, consistent with those in d–f.

NMDS ordination based on Bray–Curtis distance indicated that the compositions of plant and soil bacterial and fungal communities varied with time since restoration (Figure 1f–h). ANOSIM further demonstrated that the compositions of plant and microbial communities among plots with different restoration durations were significantly different (Figure 1f–h). The differences in plant and microbial communities between restored and natural ecosystems decreased nonlinearly with increasing time since restoration (Figure 2a–c). Nevertheless, ANOSIM showed that the compositions of the plant and microbial communities in the restored ecosystems remained significantly different from those in the natural ecosystems throughout the 53‐year restoration process, with the exception of fungal communities restored for 20 and 23 years (Appendix S1: Table S1).

**FIGURE 3:**
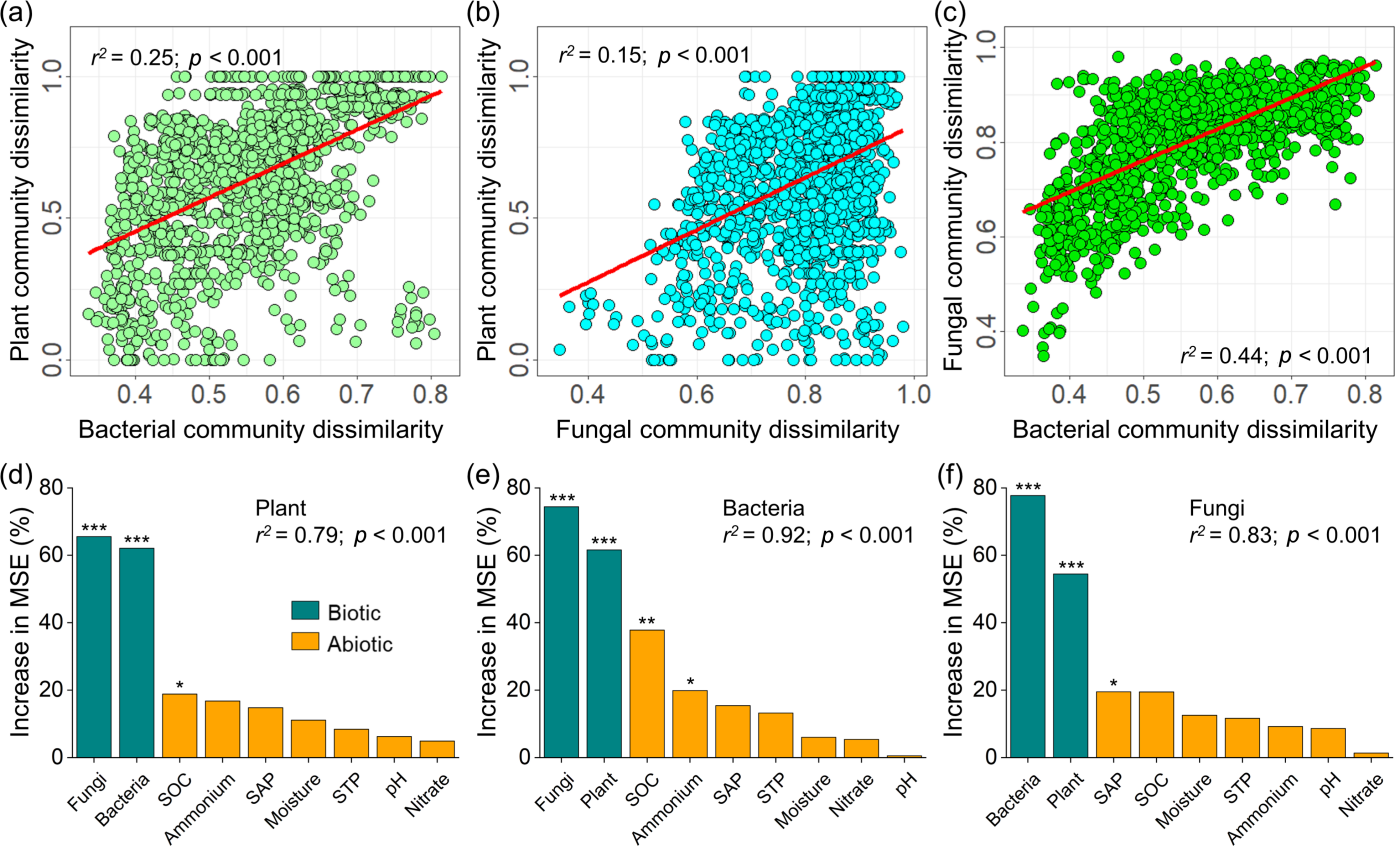
Relationships between plant and soil microbial community composition and their main predictors. Relationships between plant and soil bacterial and fungal community dissimilarity based on the Mantel test (a–c). The red fitted lines are from nonlinear regression. Random Forest mean predictor importance (increase in Mean Squared Error) of biotic (the other two community structures) and abiotic (soil environmental variables) factors on plant (d), soil bacterial (e), and fungal (f) community structures. Significant *p* values are represented by *** when *p* < 0.001, ** when *p* < 0.01, and * when *p* < 0.05. SAP, soil available phosphorus; SOC, soil organic carbon; STP, soil total phosphorus.

Mantel test further unveiled the significant positive relationships between the compositions of plant, soil bacterial and fungal communities (Figure 3a–c). Random Forest models indicated that 79%, 92%, and 83% of the variations in plant and soil bacterial and fungal community compositions, respectively, were explained by the other two community structures and soil environment variables (Figure 3d–f). Moreover, any two of the three communities (i.e., plant, soil bacteria and fungi) played pivotal roles in influencing the composition of the third community compared with soil environmental variables (Figure 3d–f), underscoring the strong interdependence in the structures of plant and soil microbial communities.

### Indicator species and thresholds of plant and soil microbial community succession

By measuring the cumulative responses of *z*− and *z*+ indicator species to restoration duration, we identified temporal threshold zones of 2–15.5 years for plants, 0.5–8.5 years for bacteria, and 2–8.5 years for fungi (Figure 2d–f). At the species level, 9 plant species, 2117 bacterial OTUs, and 366 fungal OTUs increased in relative abundance with restoration duration, whereas 2 plant species, 1467 bacterial OTUs, and 57 fungal OTUs decreased (Appendix S1: Figures S3–S5). Among these taxa, two *z*− plants (*Corispermum patelliforme* and *A. squarrosum*) belonged to Chenopodiaceae, while *z*+ plants were mainly from Poaceae and Compositae (Appendix S1: Figure S4; Table S2). Most of the *z*− indicator bacterial OTUs belonged to Actinobacteria, Proteobacteria, Bacteroidetes, and Firmicutes. Meanwhile, *z*+ bacteria exhibited higher proportions in Actinobacteria, Proteobacteria, Chloroflexi, Acidobacteria, and Cyanobacteria (Appendix S1: Figures S5 and S6). Both *z*− and *z*+ fungi were mainly from the dominant phyla, Ascomycota and Basidiomycota, with *z*− fungi accounting for a smaller proportion (Appendix S1: Figures S5 and S6).

**FIGURE 4:**
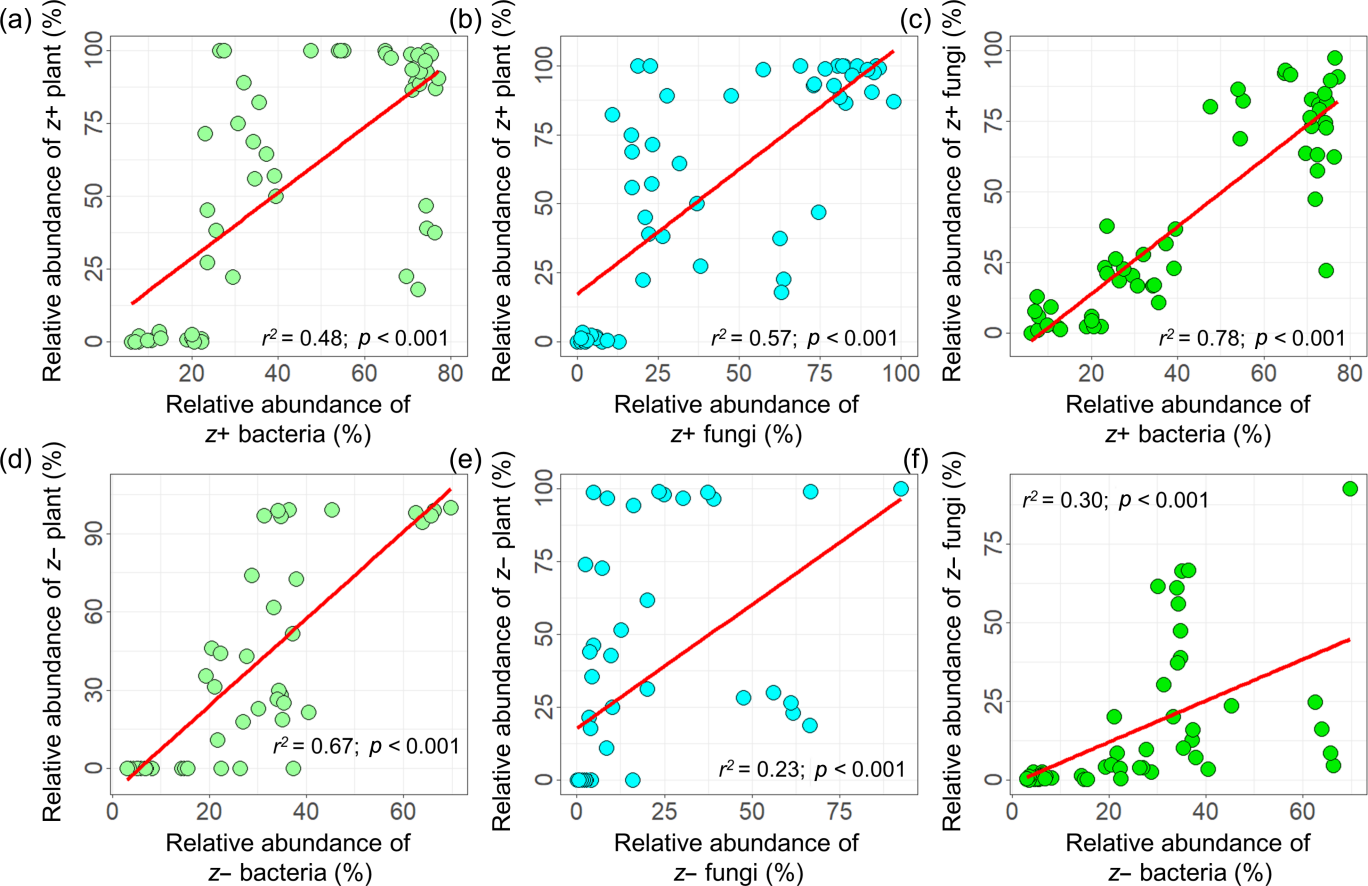
Relationships between the relative abundance of indicator species. Relationships between the relative abundance of *z*+ (a–c) and *z*− (d–f) indicator plant species, soil bacterial operational taxonomic units (OTUs), and fungal OTUs. The red fitted lines are from nonlinear regression.

Therefore, the relative abundance of dominant taxa including these indicator OTUs showed different temporal trends. The relative abundance of Actinobacteria, Chloroflexi, Acidobacteria, Cyanobacteria, and Ascomycota increased nonlinearly, whereas that of Bacteroidetes and Firmicutes decreased nonlinearly with the duration of restoration (Appendix S1: Figure S7). In addition, we found significant positive relationships among the relative abundances of *z*+ indicator species and among those of *z*− indicator species within plant and soil microbial communities (Figure 4). Similarly, the relative abundances of more plant species and soil microbial OTUs increased with rising soil nutrient content, indicating a prevalence of positive indicator species in response to soil nutrient content (Appendix S1: Table S3).

### Correlation network of indicator species in plant and soil microbial communities

**FIGURE 5:**
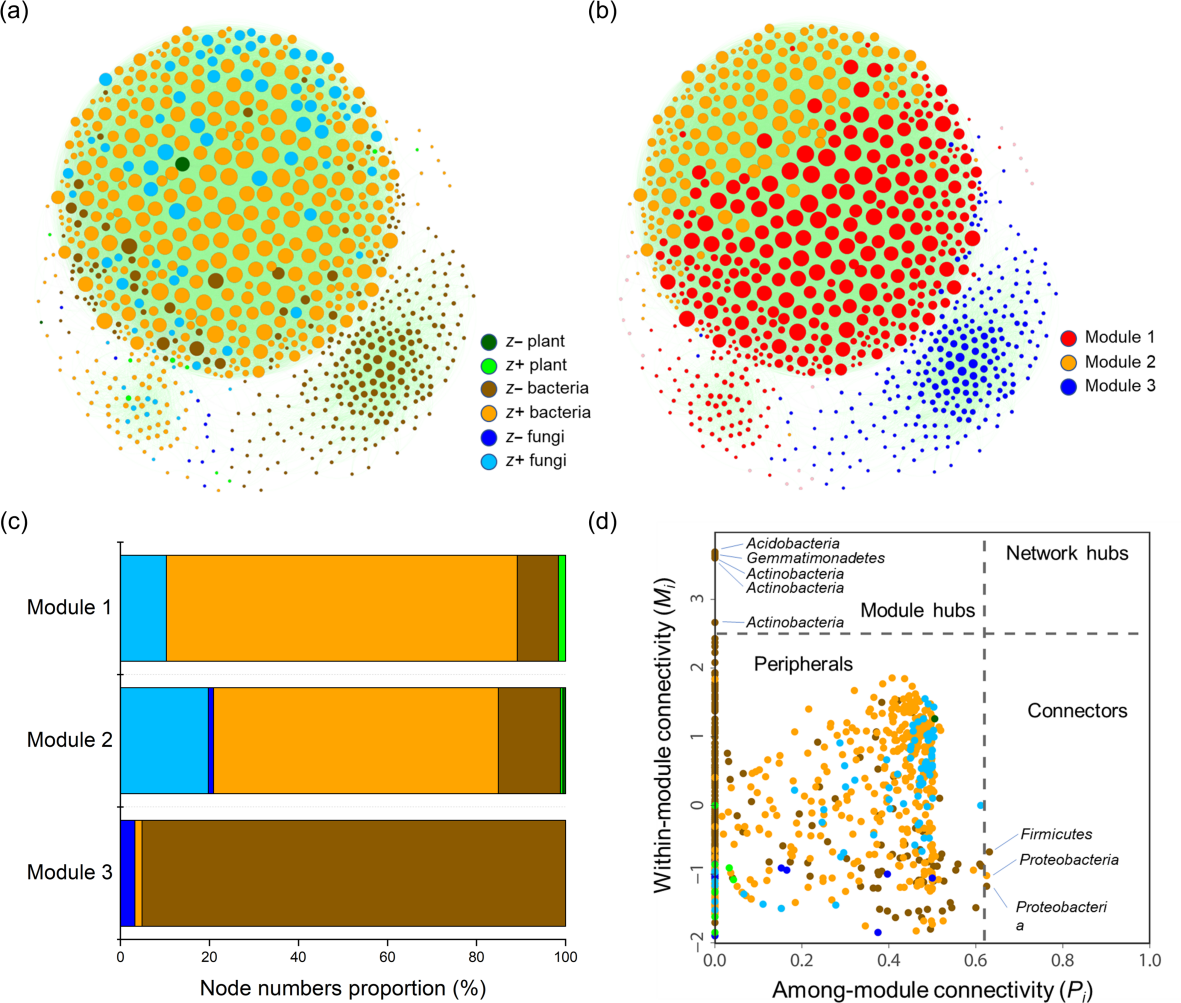
Correlation network analyses of indicator species. Correlation network including *z*− and *z*+ indicator species of plant and soil microbial communities (a). Indicator species groups are indicated by node colors. The size of each node is proportional to its connectivity, which is the sum of the connection strengths (|*ρ*|) of each node with other connected nodes. A connection represents a robust correlation (|*ρ*| > 0.6 and FDR‐corrected *p* < 0.001). Positive and negative connections are indicated by green and gray edges, respectively. Three main modules in the network indicated by node colors (b). Composition of the three main modules (c). *M*–*P* plot showing the distribution of nodes based on their topological roles (d). Each dot represents a node. Connectors and module hubs are labeled at the phylum level.

The correlation network including indicator species and soil environmental variables, revealed stronger connections among indicator species compared with those between indicator species and environmental variables based on the Mann–Whitney *U* test (Appendix S1: Figure S8). In another correlation network focusing solely on indicator species, positive connections (88.5%) dominated, overshadowing negative connections (11.5%) (Figure 5a). The network was divided into three main modules, accounting for 46.4%, 21.1%, and 30.3% of the total nodes (Figure 5b). Module 1 primarily consisted of *z*+ bacterial OTUs (78.8%), a small number of *z*+ fungal OTUs (10.3%), and six *z*+ indicator plant species (1.6%) (Figure 5c). Module 2 featured *z* +bacterial (64.0%) and fungal (19.8%) OTUs, as well as a *z*− (*A. squarrosum*, 0.6%) and a *z*+ (*A. ordosica*, 0.6%) indicator plant species (Figure 5c). The nodes of module 3 predominately consisted of *z*− bacterial OTUs (95.1%) (Figure 5c). The relative abundances of modules 1 and 2 increased significantly with restoration duration and were positively correlated with soil nutrient contents, with SOC and SAP being the most important predictors, respectively (Appendix S1: Figure S10). In contrast, the relative abundance of module 3 sharply decreased at the beginning of restoration and showed no significant relationships with any environmental variables (Appendix S1: Figure S10). The topological roles of nodes were determined by calculating their within‐module connectivity (*M*_*i*_) and among‐module connectivity (*P*_*i*_). The results showed that the majority of nodes (99.0%) were peripherals, three nodes (0.4%) were connectors, five nodes (0.6%) were module hubs, no network hubs were found, and the latter three were considered keystone nodes (Figure 5d). Seven of the eight keystone nodes were identified as *z*− indicator bacterial OTUs, and one as a *z*+ indicator bacterial OTU.

A similar network analysis was performed when assessing indicator species of soil microbial communities at the phylum level (Appendix S1: Figure S11). In networks based on positive Spearman correlations, the relative abundances of certain plant species (e.g., *A. ordosica* and *Eragrostis pilosa*) and soil microbial phyla (e.g., Acidobacteria, Chloroflexi, Cyanobacteria, and Ascomycota) responded positively to restoration duration. These taxa exhibited higher expected importance than other taxa and were strongly associated with environmental variables (SOC and SAP). However, in the negative correlation network, plant species (i.e., *A. squarrosum*) and soil microbial phyla (i.e., Bacteroidetes and Firmicutes) played more important roles than other taxa and were weakly linked to environmental variables.

### Relative importance of ecological processes

**FIGURE 6:**
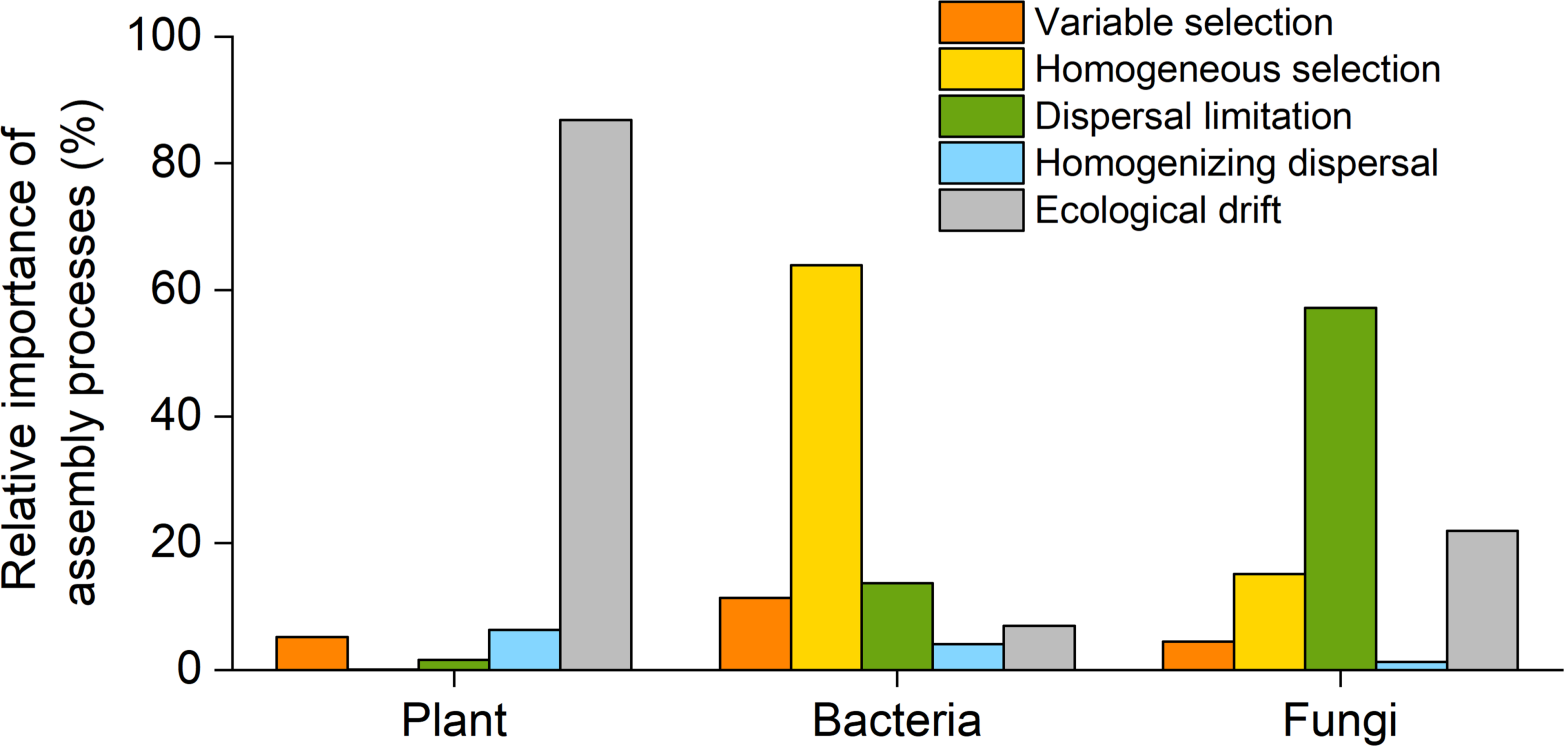
Relative importance of ecological processes mediating the community assembly of plants, soil bacteria, and fungi throughout the restoration process. The relative importance of five ecological processes (i.e., variable selection, homogeneous selection, dispersal limitation, homogenizing dispersal, and ecological drift) is estimated following the null‐modeling‐based analytical framework.

Throughout the entire restoration process, plant communities were greatly influenced by stochastic processes, particularly ecological drift (Figure 6). Ecological drift enabled by dispersal limitation played a dominant role in shaping soil fungal community structures, whereas homogeneous selection mainly contributed to soil bacterial community succession (Figure 6). Within each restoration stage, plant communities were primarily assembled by stochastic processes, including homogenizing dispersal and ecological drift (Appendix S1: Figures S12 and S13). In the case of the soil microbial community, stochastic processes were more pronounced in the mobile sandy land (Appendix S1: Figure S12). After the establishment of SCBs, the relative importance of stochastic processes controlling community succession decreased for bacteria and increased for fungi with restoration duration (Appendix S1: Figures S12 and S13). Strong negative and positive relationships between the relative importance of stochasticity and SOC were observed for bacteria and fungi, respectively (Appendix S1: Table S4).

**FIGURE 7:**
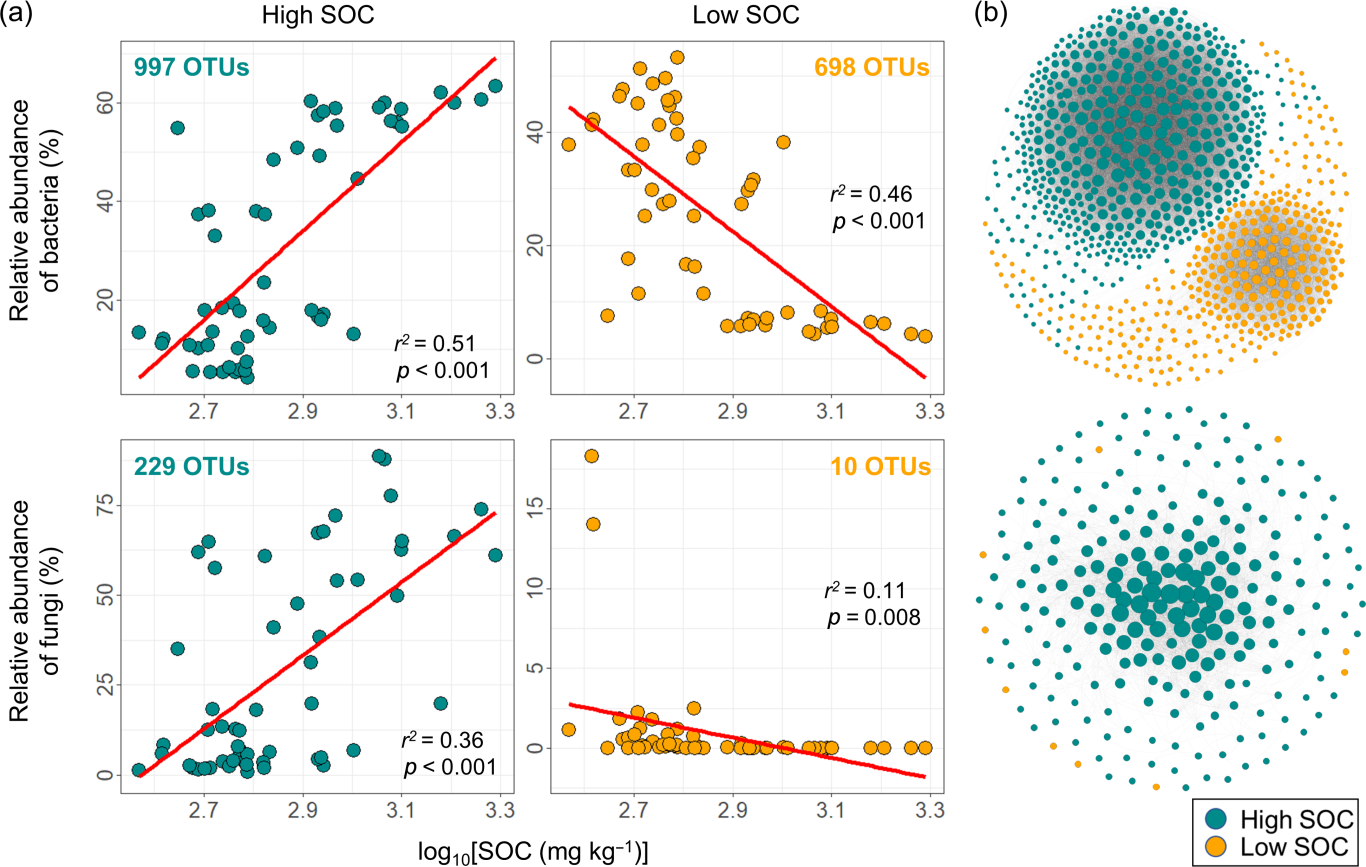
Identified habitat preferences for indicator bacterial and fungal operational taxonomic units (OTUs) in response to soil organic carbon (SOC). The *z*+ and *z*− indicator bacterial and fungal OTUs are assigned to two ecological clusters (i.e., high SOC and low SOC), respectively. The number of OTUs at the phylum level is shown in Appendix S1: Figure S14. Relationships between the relative abundance of ecological clusters and SOC (a). The red fitted lines are from linear regression. Correlation networks with nodes (indicator OTUs) colored according to ecological cluster (b). The size of each node is proportional to its connectivity. A connection indicates a robust and positive correlation (*ρ* > 0.6 and FDR‐corrected *p* < 0.001).

Given the likelihood that SOC was the most important variable for soil microbial community assembly, *z*+ and *z*− indicator OTUs in response to SOC were grouped into two ecological clusters sharing environmental preferences for (1) high SOC and (2) low SOC (Figure 7a). There were 698 *z*− and 997 *z*+ indicator bacterial OTUs, accounting for 25.1% and 30.5% of the total sequences, respectively. Additionally, there were 229 *z*+ indicator fungal OTUs, accounting for 29.2% of the total sequences, but only 10 *z*− indicator fungal OTUs, accounting for just 0.9% of the total sequences (Figure 7a; Appendix S1: Figure S14). The strong relationships between the relative abundances of the two ecological clusters and SOC indicated that these clusters were well‐defined (Figure 7a). Bacterial nodes within the same ecological clusters were more closely connected than those in different clusters, suggesting that bacterial OTUs sharing environmental preferences (high SOC and low SOC) tended to co‐occur, whereas fungal OTUs did not exhibit this pattern (Figure 7b).

## DISCUSSION

Ecosystems that have been severely degraded or damaged, such as those undergoing desertification due to wind erosion, extreme drought, and poor soil conditions, often struggle to naturally recover to their original state (Scheffer et al., 2001). However, building upon successful sand‐control experiences in China and supported by our empirical results, the establishment of SCBs emerges as a potent strategy for promoting the restoration of desertified ecosystems. Based on simultaneous surveys of plant and soil microbial communities along an exceptionally long and well‐documented chronosequence of restored desert ecosystems following the establishment of SCBs, this study uncovered both positive and negative bidirectional shifts in plant and microbial community compositions over the restoration period and identified key temporal thresholds associated with these shifts. The results underscored the significant driving effects of plant‐bacteria‐fungi correlations and SOC on temporal variations in plant and soil microbial communities. Finally, the data revealed differing assembly processes that govern succession of plant and soil microbial communities.

Previous studies have confirmed the importance of plant‐bacteria‐fungi correlations in shaping the dynamics of plant and soil microbial communities (Knelman et al., 2012; Reynolds et al., 2003). Our study supports this perspective, demonstrating that the relationships between plants and soil microbes are more influential to the succession of plant and soil microbial communities than soil environmental variables at both the community and species levels during the restoration of desert ecosystems (Figure 3; Appendix S1: Figure S8). Soil microbes have gained widespread attention in both short‐ and long‐term restoration efforts due to their critical roles in maintaining ecosystem functions and restoring degraded ecosystems (Liao et al., 2023; Nemergut et al., 2007; Sha et al., 2023). As pioneers, they undergo succession in nonvegetated soils before plant colonization (Nemergut et al., 2007). Consequently, soil microbes responded rapidly to the establishment of SCBs, with notable positive changes in community composition, as evidenced by an increase in positive soil microbial OTUs, occurring within the initial 0.5–8.5 years of restoration. In contrast, plant communities showed delayed compositional shifts, occurring between 2 and 15.5 years. These results further demonstrate that the composition and structure of microbial communities are more sensitive to active restoration efforts than are plant communities (Kardol et al., 2006; Li et al., 2000; Wubs et al., 2016). This discrepancy in response is not surprising, given the vastly different life cycles of microbes (measured in hours) compared with plant species (ranging from one to many years).

Throughout the restoration chronosequence, positive (*z*+) indicator species in both plant and soil microbial communities were more prevalent than negative (*z*−) indicator species. This pattern reflects the ongoing processes of sand stabilization and soil development. More importantly, soil nutrient contents, particularly SOC, were strongly correlated with two positive biological groups (modules 1 and 2) within the correlation network. SOC, a pivotal component of soil fertility, gradually accumulates through the decomposition of straw and plant litter during ecological restoration. This accumulation of SOC creates more favorable conditions and broader niches for species colonization (Drenovsky et al., 2004; Dybzinski et al., 2008; Poorter et al., 2021). Soil available nitrogen and phosphorus, which are crucial limiting resources in drylands, exert a profound influence on the structure and function of plant and soil microbial communities (Delgado‐Baquerizo et al., 2013; Tang et al., 2016). In contrast to modules 1 and 2, module 3 was mostly composed of negative soil bacterial OTUs and showed a steady decline in relative abundance over restoration duration. This module represents the initial microbial community involved in ecological restoration. The sharp decline in the relative abundance of module 3 is mainly attributed to the following two aspects: (1) changes in the original soil microenvironment (e.g., mobile sandy land) induced by SCBs and human activities (laying straw) and (2) changes in plant community composition. The transplantation and establishment of more plants increased organic matter input, providing additional niches for positive soil microbes. This further suppressed the dominance of negative soil microbial OTUs in module 3 through competition (Figure 5). The negative response of biological groups within module 3 may contribute both to species replacement of plant and soil microbial communities at the early stage of restoration and to the diversification of the subsequent community, thereby influencing the structure and function of ecological networks (Reynolds et al., 2003; Wubs et al., 2019). This elucidates why the keystone nodes predominately consisted of negative bacteria (Figure 5d).

Due to the combined influence of SCBs, local climatic conditions, and species pools, substantial changes in plant and soil microbial communities reduced the differences from natural communities within 15.5 and 8.5 years of restoration (Hodkinson et al., 2003). However, even after 53 years of restoration, plant and soil microbial communities still differed significantly from their corresponding natural communities. This persistent dissimilarity may be due to the complexity of restoring community composition, including factors such as species identity and relative abundance, which are constrained by dispersal and recruitment limitations (Poorter et al., 2021). The differences in dominant plant and soil microbial taxa with time since restoration can be explained by their life‐history strategies and functions (Appendix S1: Figure S7). For example, Actinobacteria actively promote the decomposition of soil organic matter (Heuer et al., 1997). Chloroflexi and Cyanobacteria, being photoautotrophic organisms, can improve soil nutrient status through photosynthesis (Shih Patrick et al., 2017; Zumsteg et al., 2012). Meanwhile, the plant family Chenopodiaceae and the bacterial phylum Firmicutes exhibit high adaptability to extremely arid environments.

Generally, ecological succession is expected to follow a predictable path, starting from pioneer species and progressing toward a climax community. However, our results unexpectedly showed that stochastic processes played a more important role in plant community assembly compared with deterministic processes. This outcome can be explained by the following reasons: (1) the restored plant communities were dominated by the drought‐tolerant *A. ordosica*, which adapts to the stressful conditions and further inhibits the colonization of other species through its strong allelopathy (Deng et al., 2016; Yang et al., 2012). This dominance leads to a small species pool and low abundance of other species; (2) and thus, the restricted species pool (with only 28 species observed) leads to an overestimation of stochasticity during plant community succession based on the null model (Chase & Myers, 2011; Gao et al., 2020), despite observable changes in plant community composition with increasing restoration duration (Figures 1 and 2). Acknowledging this, future studies should incorporate more empirical data from relevant restoration studies to validate and refine these findings.

The frequent movement of shifting sand in mobile sandy land provides ample opportunities for soil microbes to migrate, making stochastic processes more influential in shaping soil microbial communities (Dini‐Andreote et al., 2015; Ferrenberg et al., 2013). The establishment of SCBs helps stabilize the soil by fixing shifting sand and enhancing soil nutrients (Li et al., 2007), thereby creating a uniform selection pressure on soil microbial communities. This effect resembles the pattern observed in soil microbial communities after wildfire disturbance (Ferrenberg et al., 2013; Liang et al., 2020). Interestingly, homogeneous selection became increasingly important for soil bacteria but less relevant for soil fungi with time since restoration, with stochasticity showing the opposite pattern. The positive relationship between SOC and stochasticity in fungal community succession aligns with previous studies, indicating that stochastic effects increase with higher resource availability (Chase, 2010; Liang et al., 2020; Zhou et al., 2014). Nearly all fungal OTUs (95.82%) preferred high SOC environments, suggesting that increased SOC promotes the emergence and growth of more fungal lineages, thereby enhancing stochasticity. In contrast, the negative relationship between SOC and stochasticity for bacteria indicates that higher SOC can act as a selective pressure on bacterial communities. The nonrandom co‐occurrence pattern observed in the correlation network further underscores the dominance of deterministic processes (Barberán et al., 2012; Horner‐Devine et al., 2007). The evident niche differentiation (i.e., different preferences for SOC) during bacterial community succession can be explained by the following two reasons: (1) bacterial OTUs are more profoundly influenced by restoration duration and changes in plant community compared with fungi (Figure 4; Appendix S1: Figure S15); and (2) bacterial communities may experience stronger competition for C resources than fungal communities (Ballhausen & de Boer, 2016; de Vries et al., 2018; Fuchslueger et al., 2014; Morriën et al., 2017). As a result, the homogeneous selection imposed by niche differentiation leads to less phylogenetic turnover in bacterial community composition than expected by the null model, whereas the phylogenetic turnover in fungal community composition, dominated by stochasticity, showed little deviation from the null expectation.

In this study, using the space‐for‐time substitution approach to compare different plots with varying time since restoration is both reasonable and reliable. Firstly, all the plots were located in the southern margin of the Tengger Desert, sharing the same climatic conditions, and the SCBs were established under similar initial environmental conditions in the mobile sandy land. Therefore, there are no substantial spatial differences in climate variables and the initial soil status that warrant concern in our study. Secondly, the variations observed in plant and soil microbial community composition in this scenario are attributed primarily to the age of SCBs (i.e., restoration duration). Furthermore, due to the difficulty of obtaining long‐term continuous data from the same plot, the space‐for‐time substitution approach is widely employed in chronosequence studies of vegetation succession and soil development and is generally considered to yield reliable results (Hasselquist et al., 2015; Pickett, 1989; Walker et al., 2010). However, it is essential to note that a time series approach should be preferred for increased precision whenever the data are available.

We believe that our analyses of plant and soil microbial community succession offer significant practical implications. First, fixing shifting sands should be prioritized as the initial and most important step in restoring desertified ecosystems. Second, our results identified key plant and microbial indicator species along the restoration timeline, which serve as early signs of changes in desertified ecosystems. Although the predominance of positive indicator species in a local community generally suggests a healthy ecosystem, a shift toward community dominance by negative indicator species may indicate a degraded ecosystem. Therefore, monitoring indicator species in natural ecosystems can help identify early warning signs of desertification (Maestre et al., 2016). Third, our analyses showed that positive indicator species play an important role in maintaining biodiversity and promoting desert ecological restoration. These species can guide the selection and introduction of suitable plant species (e.g., *A. ordosica*) and the production of microbial inoculants (e.g., Cyanobacteria, which are key component of biocrusts). Utilizing these species can aid in plant community reconstruction and expedite desert ecosystem restoration (Lan et al., 2014; Lázaro‐González et al., 2023). Overall, incorporating the scientific insights gained from this study into practical applications is paramount for addressing the challenges posed by desertification and promoting sustainable development in drylands.

## AUTHOR CONTRIBUTIONS

Jianming Deng conceived the study. Field data were collected by Qingqing Hou, Weigang Hu, Qiajun Du, and Ying Sun. Laboratory measurements were performed by Qingqing Hou, Ying Sun, Junlan Xiong, Longwei Dong, Shuran Yao, Jie Peng, Yuan Sun, Muhammad Aqeel, Muhammad Adnan Akram, Rui Xia, Yahui Zhang, Xiaoting Wang, Shubin Xie, Liang Zhang, Fan Li, Yan Deng, Jiali Luo, and Jingyan Yuan. Statistical analysis and data integration were carried out by Qingqing Hou and Weigang Hu with suggestions and help from Jinzhi Ran and Jianming Deng. The first draft was written by Qingqing Hou, Weigang Hu, and Jianming Deng. Elly Morriën, Qiang Yang, Karl J. Niklas, Muhammad Aqeel, Liang Wang and Quanlin Ma contributed to the writing of the manuscript. All authors contributed to the final version of the manuscript.

## CONFLICT OF INTEREST STATEMENT

The authors declare no conflicts of interest.

## Supporting information


Appendix S1:

## Data Availability

Data and code (Hou et al., 2024) are available in Figshare at https://doi.org/10.6084/m9.figshare.20859589. The raw sequence data are available in the National Center for Biotechnology Information's Sequence Read Archive under BioProject accession numbers PRJNA877077 and PRJNA877300 (https://www.ncbi.nlm.nih.gov/bioproject/PRJNA877077 and https://www.ncbi.nlm.nih.gov/bioproject/PRJNA877300) for bacteria and fungi, respectively.
