# Supplementary material for: Active restoration efforts drive community succession and assembly in a desert during the past 53 years
Source: Ecol Appl. 2024 Nov 25;35(1):e3068. doi: 10.1002/eap.3068 (PMC11725625; doi:10.1002/eap.3068)
Supplement: Supplementary file 1 — Appendix S1: [file EAP-35-e3068-s001.pdf]

Ecological Applications

Appendix S1 for

Active restoration efforts drive community succession and assembly in a desert during the past 53 years

Qingqing Hou, Weigang Hu, Ying Sun, Elly Morriën, Qiang Yang, Muhammad Aqeel, Qiajun Du, Junlan Xiong, Longwei Dong, Shuran Yao, Jie Peng, Yuan Sun, Muhammad Adnan Akram, Rui Xia, Yahui Zhang, Xiaoting Wang, Shubin Xie, Liang Wang, Liang Zhang, Fan Li, Yan Deng, Jiali Luo, Jingyan Yuan, Quanlin Ma, Karl J. Niklas, Jinzhi Ran, Jianming Deng

Section S1

Threshold indicator species analyses

Threshold indicator species analysis (TITAN) using the R package "TITAN2" was employed to identify negative and positive indicator species of plant and soil microbial communities in response to restoration duration and environmental variables, and to calculate their thresholds (Baker & King, 2010). For each species, two indicator value (IndVal) scores were calculated by integrating the relative abundance and occurrence frequency within each group in a two-group classification. Species with an IndVal score of 100% appeared in all samples within one temporal and environmental gradient group and not in other groups. IndVal scores were used to identify change points along temporal and environmental gradients. Midpoints between two temporal and environmental gradient values were used as candidate change points to iteratively split the gradients into two groups, thereby generating two indicator scores at each split. The relative magnitude of the indicator scores for the groups on each side of a candidate change point reflects whether a species responds negatively or positively to restoration duration and environmental variables (Baker & King, 2010). Among candidate splits, any temporal or environmental gradient that results in an IndVal maximum was considered the threshold for that species (Baker & King, 2010). IndVal scores were standardized by the mean and standard deviation of randomized permutations from IndVals, resulting in indicator *z* scores. Thus, the *z* score distinguished negative (*z*-) and positive (*z*+) species responses to restoration duration and environmental variables. The sums of all *z*- and *z*+ scores were used to

assess the cumulative positive and negative responses of species within the community. The temporal or environmental gradient values corresponding to the largest sum of *z*- and *z*+ scores were identified as community-level thresholds (Baker & King, 2010). Species with < 3 occurrences were excluded, and their abundances were $\log_{10}(x+1)$ transformed.

Correlation network analyses

We constructed a correlation network including soil environmental variables and indicator species of plant and soil microbial communities in response to restoration duration. For soil bacteria and fungi, we selected *z*- and *z*+ indicator species with *z* scores in the top 20% to reduce network complexity and facilitate the identification of key taxa (Barberán et al., 2012). We calculated all possible Spearman's correlations among soil environmental variables and indicator species. Robust Spearman's correlations (ρ) with absolute values > 0.6 and FDR-corrected $P < 0.001$ were used. Nodes represent soil environmental variables and indicator species, and edges represent correlations between these nodes. The size of each node was proportional to its connectivity, which is the sum of the connection strengths ($|\rho|$) of each node with other connected nodes (Poorter et al., 2021; Zhou et al., 2011). Networks were visualized using the interactive Gephi platform. We then tested whether nodes exhibited a non-random co-occurrence pattern by fitting a power-law model to determine the degree distribution of nodes in the constructed correlation network (Banerjee et al., 2018).

A similar correlation network including only indicator species was also constructed. The nodes in the network were divided into different modules using the R package “igraph”. Nodes within a module are more closely connected to each other than to nodes in other modules (Olesen Jens et al., 2007). Each node was assigned a role based on its topological properties, described by within-module connectivity (M_i) and among-module connectivity (P_i) (Guimerà & Nunes Amaral, 2005; Olesen Jens et al., 2007). M_i reflects the connection of a node within its own module, and P_i reflects the connection of a node with nodes in other modules. Then, nodes were categorized into four types: (i) peripherals ($M_i \leq 2.5$ and $P_i \leq 0.62$), which have few links to nodes within their own modules and almost no links to nodes within other modules; (ii) connectors ($M_i \leq 2.5$ and $P_i > 0.62$), which are highly connected to nodes in other modules; (iii) module hubs ($M_i > 2.5$ and $P_i \leq 0.62$), which are highly connected to nodes within their own modules; and (iv) network hubs ($M_i > 2.5$ and $P_i > 0.62$), which act as both connectors and module hubs (Olesen Jens et al., 2007; Zhou et al., 2011). The latter three categories are defined as keystone nodes, playing critical roles in maintaining network stability and driving ecosystem functions (Banerjee et al., 2018; Guimerà & Nunes Amaral, 2005; Olesen Jens et al., 2007; Zhou et al., 2011). Additionally, we constructed networks based on positive ($\rho > 0.6$) and negative ($\rho <$ 0.6) correlations among indicator species and soil environmental variables when assessing soil indicator species at the phylum level.

Estimating importance of ecological processes

We inferred the relative contributions of deterministic (i.e., variable selection and homogenous selection) and stochastic (i.e., dispersal limitation coupled with drift, homogenizing dispersal and drift acting alone) processes to plant and soil microbial community assembly through a null-modeling-based quantitative framework developed by Stegen et al. (2012, 2013, 2015). The framework considers both phylogenetic and taxonomic turnover between communities.

We calculated the β-mean nearest taxon distance (βMNTD) to characterize the pairwise phylogenetic turnover between communities. βMNTD is the abundance-weighted mean phylogenetic distance between each OTU in one community (*k*) and its closest relative in a second community(*m*):

$$\beta\text{MNTD} = 0.5\left[\sum_{i_k=1}^{n_k} f_{i_k} \min\left(\Delta_{i_k j_m}\right) + \sum_{i_m=1}^{n_m} f_{i_m} \min\left(\Delta_{i_m j_k}\right)\right] \quad (1)$$

where fi_k is the relative abundance of OTU i in community k, n_k is the number of OTUs in community k and $\min(\Delta i_k j_m)$ is the minimum phylogenetic distance between OTU i in community k and all OTUs j in community m.

A null distribution of βMNTD was calculated by repeating the randomization 999 times after a randomization was used to shuffle species names and abundances across the tip of the phylogeny. To quantify the degree to which βMNTD deviates from the null model expectation, we used the β-nearest taxon index (βNTI), which measures the number of standard deviations of the observed βMNTD from the mean of the null distribution (Stegen et al., 2012, 2013):

$$\beta\text{NTI} = \left(\beta\text{MNTD}_{\text{observed}} - \overline{\beta\text{MNTD}_{\text{null}}}\right) / \text{sd}\left(\beta\text{MNTD}_{\text{null}}\right) \quad (2)$$

|βNTI| > 2 indicates a significant deviation from the expected phylogenetic turnover, implying that deterministic processes primarily control the observed turnover between a pair of communities (Stegen et al., 2012, 2013). Specifically, βNTI < -2 or > +2 denote that homogenous selection or variable selection caused the similarities or differences between a pair of communities, respectively. In contrast, |βNTI| < 2 indicates that stochastic processes, including dispersal limitation coupled with drift, homogenizing dispersal, and ecological drift alone, drive the observed turnover (Hu et al., 2022; Stegen et al., 2012, 2013).

To further distinguish these stochastic processes, we extended the Raup–Crick metric (Chase et al., 2011) to incorporate the relative abundance of species. Similar to the above, we standardized the deviation between observed Bray–Curtis and the null distribution (i.e., the expectation of drift acting alone) of Bray–Curtis values to quantify the modified Raup–Crick metric. The resulting metric varies between -1 and 1, and is referred to as the Bray–Curtis-based Raup–Crick (RC_{bray}) (Chase et al., 2011; Stegen et al., 2013). $|RC_{bray}|$ > 0.95 indicates a significant deviation from expected turnover. Specifically, ecological drift enabled by dispersal limitation and homogenizing dispersal increase and decrease compositional differences, and thus resulting in RC_{bray} values greater than +0.95 and less than -0.95, respectively. In contrast, nonsignificant RCbray values (|RCbray| < 0.95) estimate the influence of drift acting alone (Hu et al., 2022; Stegen et al., 2013).

In summary, we followed a two-step procedure to estimate the relative importance of multiple ecological processes in mediating plant and microbial

community assembly (Hu et al., 2022; Stegen et al., 2013). First, for all pairwise community comparisons, the relative importance of variable selection and homogeneous selection were estimated by calculating the fractions of pairwise βNTI values above +2 and below –2, respectively. In the second step, we quantified RC_{bray} for pairwise community comparisons not governed by selection (i.e., those with |βNTI| < 2). Within this set, the relative importance of ecological drift enabled by dispersal limitation and homogenizing dispersal were estimated as the fractions of all pairwise comparisons with RC_{bray} > +0.95 and < –0.95, respectively. The fraction of all pairwise comparisons with $|RC_{bray}|$ < 0.95 estimates the relative importance of drift acting alone (Hu et al., 2022; Stegen et al., 2013).

Section S1 References

Baker, M. E., and R. S. King. 2010. "A new method for detecting and interpreting biodiversity and ecological community thresholds." *Methods in Ecology and Evolution* **1**:25–37.

Banerjee, S., K. Schlaeppi, and M. G. A. van der Heijden. 2018. "Keystone taxa as drivers of microbiome structure and functioning." *Nature Reviews Microbiology* **16**:567–576.

Barberán, A., S. T. Bates, E. O. Casamayor, and N. Fierer. 2012. "Using network analysis to explore co-occurrence patterns in soil microbial communities." *The ISME Journal* **6**:343–351.

Chase, J. M., N. J. B. Kraft, K. G. Smith, M. Vellend, and B. D. Inouye. 2011. "Using null models to disentangle variation in community dissimilarity from variation

in α-diversity." *Ecosphere* **2**:art24.

Guimerà, R., and L. A. Nunes Amaral. 2005. "Functional cartography of complex metabolic networks." *Nature* **433**:895–900.

Hu, W., Q. Hou, M. Delgado-Baquerizo, J. C. Stegen, Q. Du, L. Dong, M. Ji, Y. Sun, S. Yao, H. Gong, J. Xiong, R. Xia, J. Liu, M. Aqeel, M. A. Akram, J. Ran, and J. Deng. 2022. "Continental-scale niche differentiation of dominant topsoil archaea in drylands." *Environmental Microbiology* **24**:5483–5497.

Olesen Jens, M., J. Bascompte, L. Dupont Yoko, and P. Jordano. 2007. "The modularity of pollination networks." *Proceedings of the National Academy of Sciences* **104**:19891–19896.

Poorter, L., D. Craven, C. Jakovac Catarina, T. van der Sande Masha, L. Amissah, F. Bongers, L. Chazdon Robin, E. Farrior Caroline, S. Kambach, A. Meave Jorge, R. Muñoz, N. Norden, N. Rüger, M. van Breugel, M. Almeyda Zambrano Angélica, B. Amani, L. Andrade José, H. S. Brancalion Pedro, N. Broadbent Eben, H. de Foresta, H. Dent Daisy, G. Derroire, J. DeWalt Saara, M. Dupuy Juan, M. Durán Sandra, C. Fantini Alfredo, B. Finegan, A. Hernández-Jaramillo, L. Hernández-Stefanoni José, P. Hietz, B. Junqueira André, K. N'dja Justin, G. Letcher Susan, M. Lohbeck, R. López-Camacho, M. Martínez-Ramos, P. L. Melo Felipe, F. Mora, C. Müller Sandra, E. N'Guessan Anny, F. Oberleitner, E. Ortiz-Malavassi, A. Pérez-García Eduardo, X. Pinho Bruno, D. Piotto, S. Powers Jennifer, S. Rodríguez-Buriticá, M. A. Rozendaal Danaë, J. Ruíz, M. Tabarelli, M. Teixeira Heitor, E. Valadares de Sá

Barretto Sampaio, H. van der Wal, M. Villa Pedro, W. Fernandes Geraldo, A. Santos Braulio, J. Aguilar-Cano, S. de Almeida-Cortez Jarcilene, E. Alvarez-Davila, F. Arreola-Villa, P. Balvanera, M. Becknell Justin, A. L. Cabral George, C. Castellanos-Castro, H. J. de Jong Ben, E. Nieto Jhon, M. Espírito-Santo Mário, C. Fandino Maria, H. García, D. García-Villalobos, S. Hall Jefferson, A. Idárraga, J. Jiménez-Montoya, D. Kennard, E. Marín-Spiotta, R. Mesquita, R. F. Nunes Yule, S. Ochoa-Gaona, M. Peña-Claros, N. Pérez-Cárdenas, J. Rodríguez-Velázquez, S. Villanueva Lucía, B. Schwartz Naomi, K. Steininger Marc, D. M. Veloso Maria, F. M. Vester Henricus, C. G. Vieira Ima, G. B. Williamson, K. Zanini, and B. Hérault. 2021. "Multidimensional tropical forest recovery." *Science* **374**:1370–1376.

Stegen, J. C., X. Lin, J. K. Fredrickson, X. Chen, D. W. Kennedy, C. J. Murray, M. L. Rockhold, and A. Konopka. 2013. "Quantifying community assembly processes and identifying features that impose them." *The ISME Journal* **7**:2069–2079.

Stegen, J. C., X. Lin, J. K. Fredrickson, and A. E. Konopka. 2015. "Estimating and mapping ecological processes influencing microbial community assembly." *Frontiers in Microbiology* **6**:370.

Stegen, J. C., X. Lin, A. E. Konopka, and J. K. Fredrickson. 2012. "Stochastic and deterministic assembly processes in subsurface microbial communities." *The ISME Journal* **6**:1653–1664.

Zhou, J., Y. Deng, F. Luo, Z. He, and Y. Yang. 2011. "Phylogenetic molecular

ecological network of soil microbial communities in response to elevated CO_2." *mBio* **2**:e00122–11.

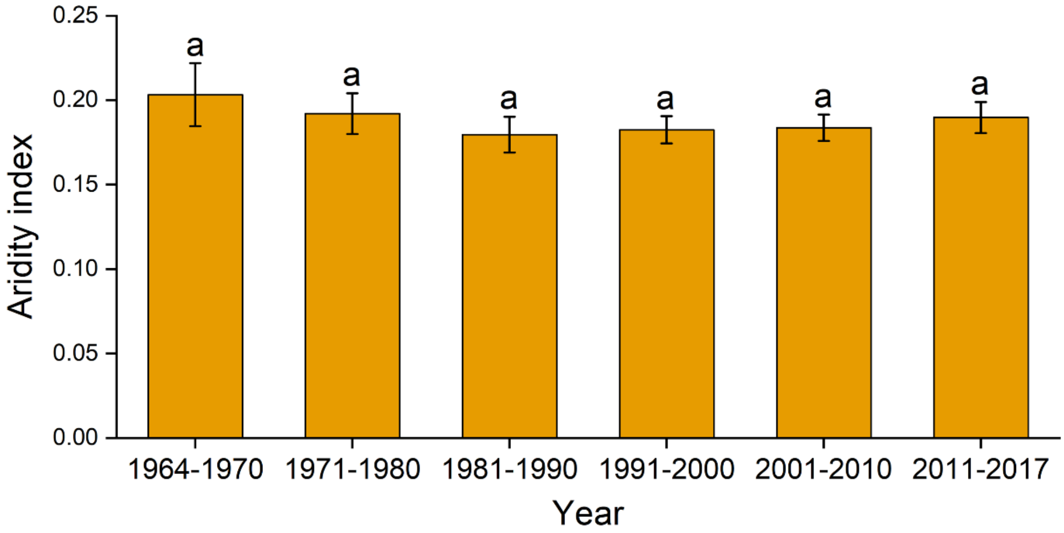

Figure S1. Aridity index from 1964 to 2017 in the research area. The same letters indicate that there is nonsignificant difference ($P > 0.05$) in aridity index at different stages based on LSD.

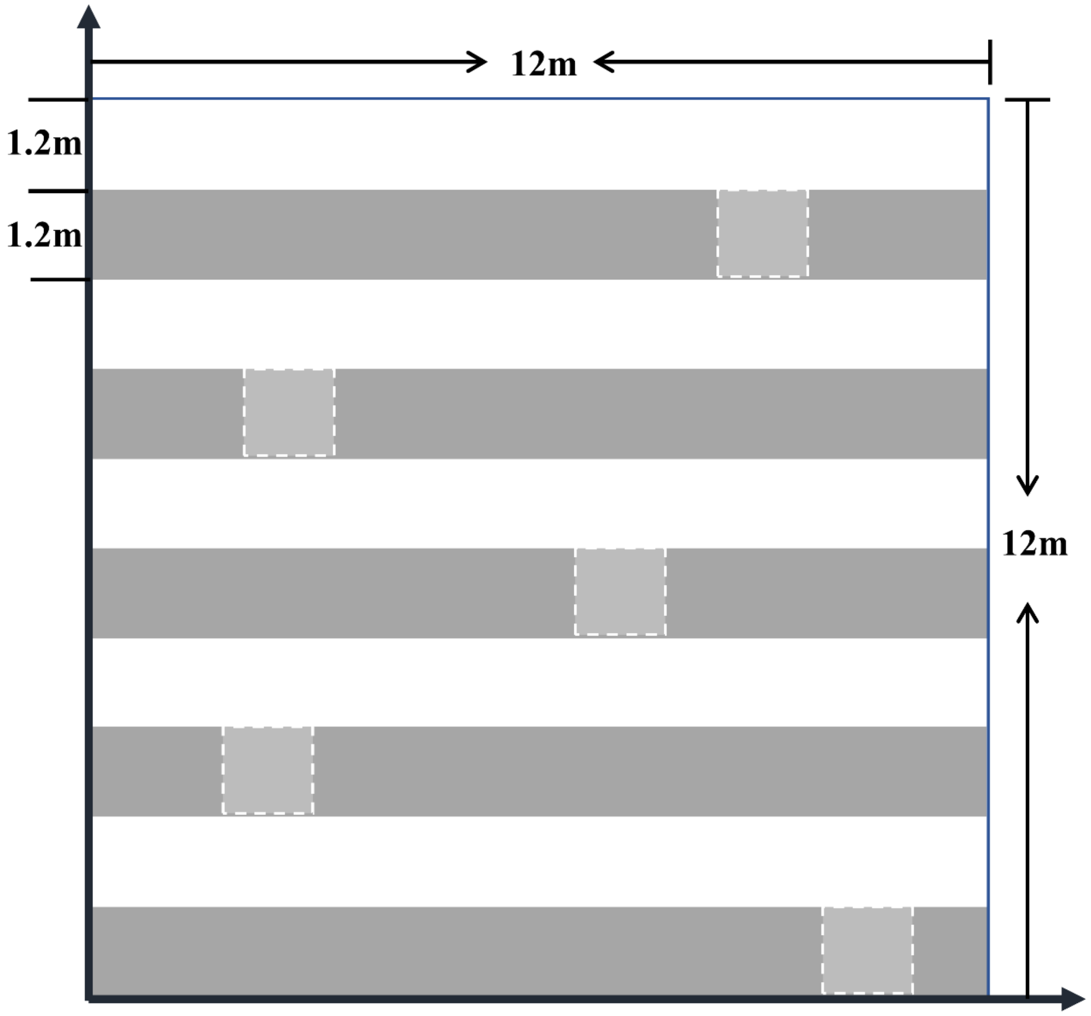

Figure S2. Scheme of a 12 × 12 m quadrat surveyed in the field. Five 12-m-long transects (dark grey shaded areas; spaced 1.2 m apart) are arranged for the survey of vegetation. The white dashed squares are five randomly selected 1.2 × 1.2 m subplots for the collection of soil samples.

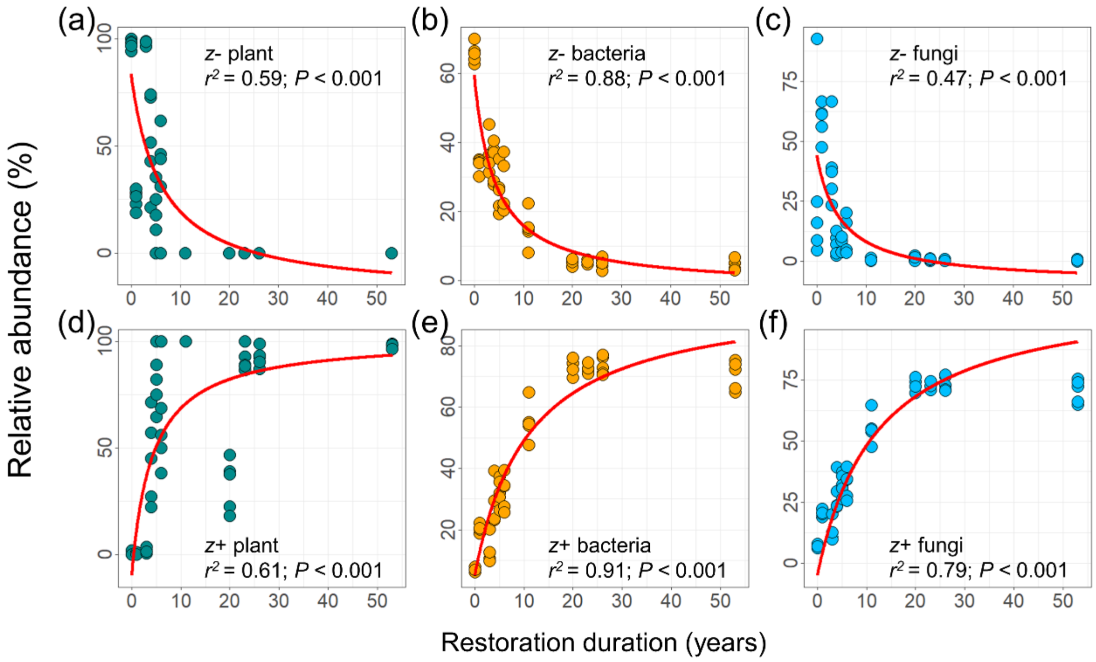

Figure S3. Relationships between SCB restoration duration and the relative abundance of *z*- and *z*+ indicator species. Relationships between SCB restoration duration and the relative abundance of *z*- and *z*+ indicator plant species (a, d), soil bacterial OTUs (b, e) and fungal OTUs (c, f). For details on the indicator species, see Figures S4–6 and Table S2. The red fitted lines are from nonlinear regression.

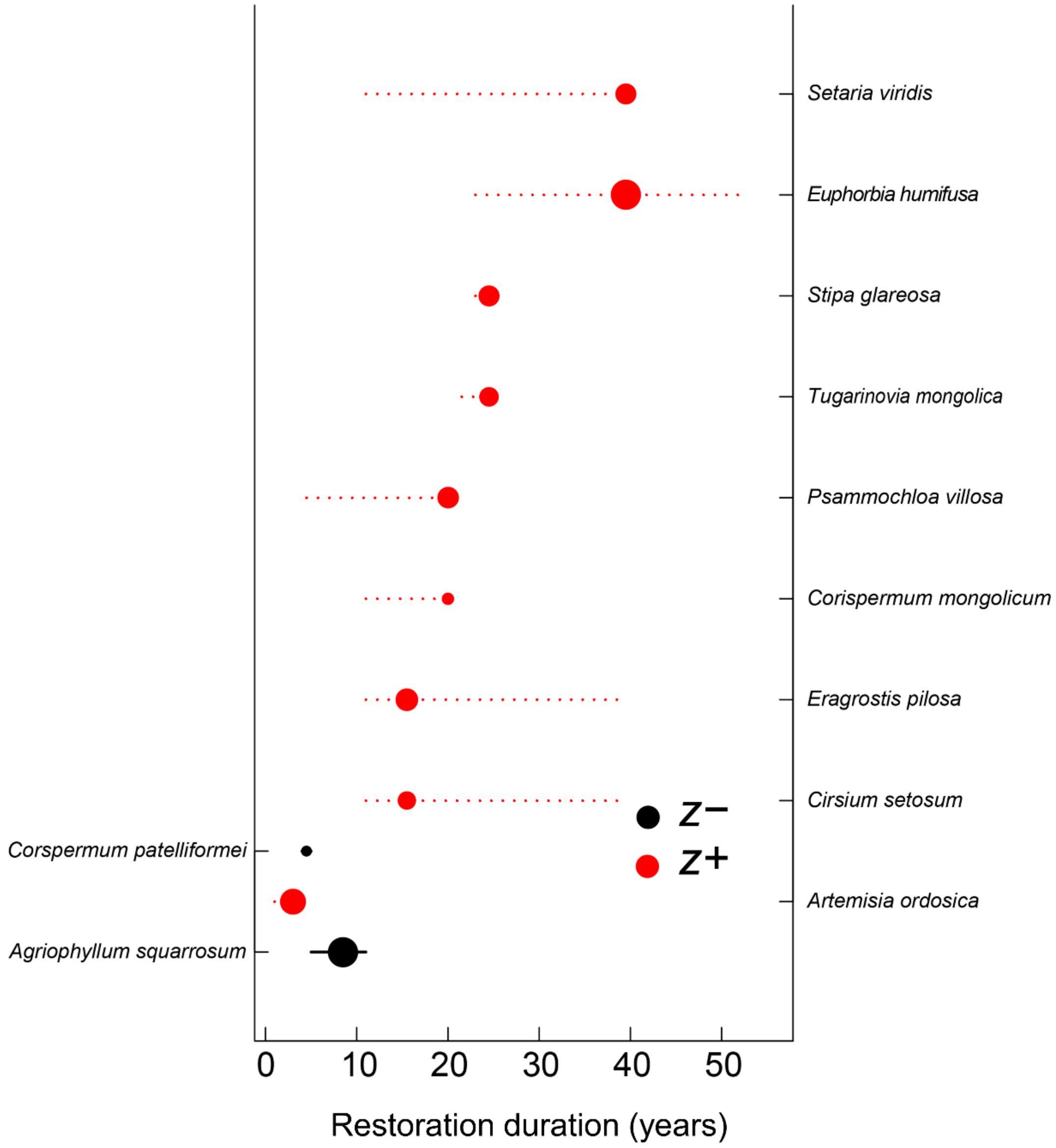

Figure S4. Threshold indicator taxa analysis of plant communities in response to SCB restoration duration. The relative abundance of individual plant species in response to SCB restoration duration. Black and red dots represent the *z*- and *z*+ responses to SCB restoration duration, respectively. Symbol size is proportional to the magnitude of the response (*z* score). Horizontal lines represent the 95% bootstrap confidence interval.

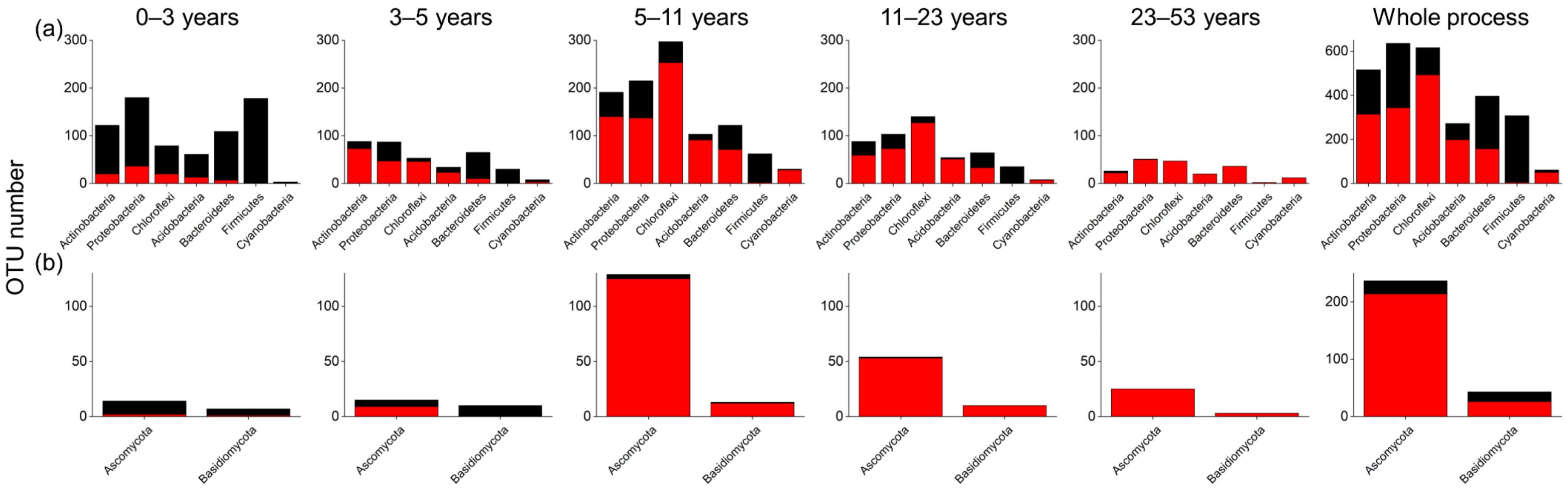

Figure S5. Threshold indicator taxa analysis of soil microbial communities in response to SCB restoration duration. The number of *z*- and *z*+ indicator OTUs in dominant bacterial (a) and fungal (b) phyla at different stages and throughout the restoration process. Black and red represent the *z*- and *z*+ responses to SCB restoration duration, respectively.

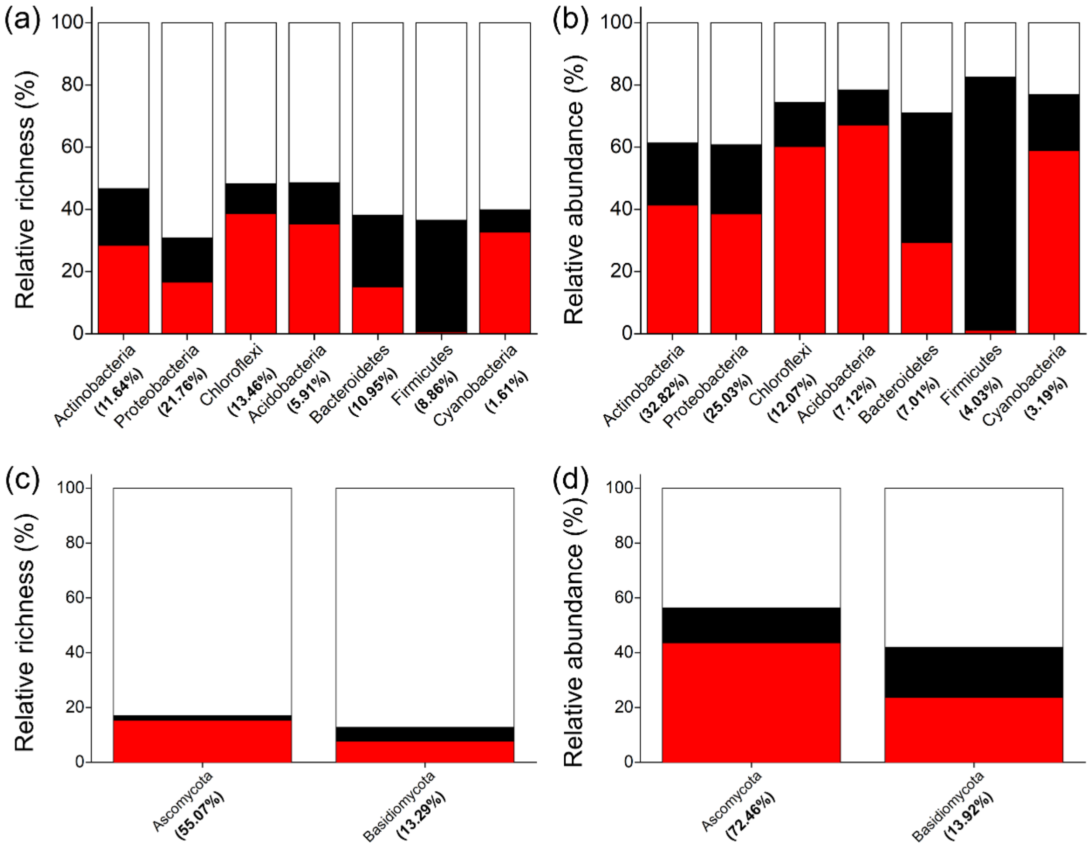

Figure S6. Relative richness and abundance of indicator OTUs in dominant phyla. Relative richness and abundance of indicator OTUs in dominant soil bacterial (a, b) and fungal (c, d) phyla. The numbers in brackets represent the relative richness and abundance of dominant phyla in the community. Black and red represent the *z*- and *z*+ responses to restoration duration, respectively.

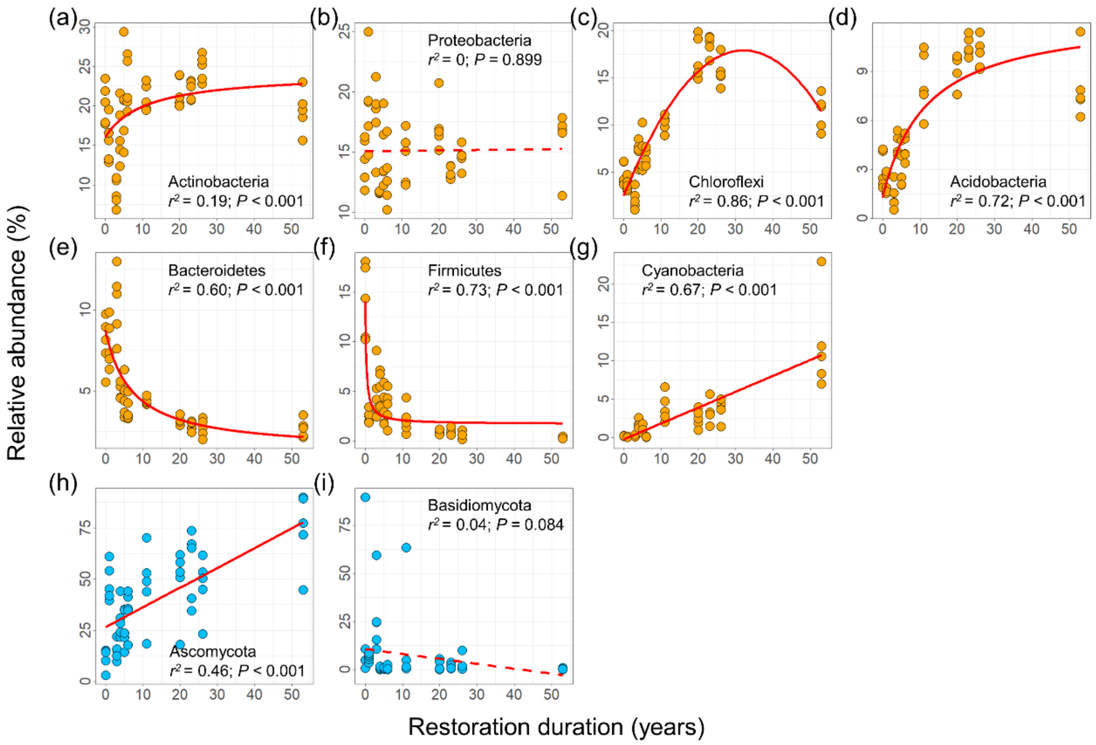

Figure S7. Relationships between SCB restoration duration and the relative abundance of indicator OTUs at the phylum level. Relationships between SCB restoration duration and the relative abundance of indicator bacterial (a–g) and fungal (h, i) OTUs at the phylum level. Only the dominant phyla are shown. The red fitted lines are from linear and nonlinear regression. The solid and dotted lines represent statistically significant ($P \leq 0.05$) and nonsignificant ($P > 0.05$) relationships, respectively.

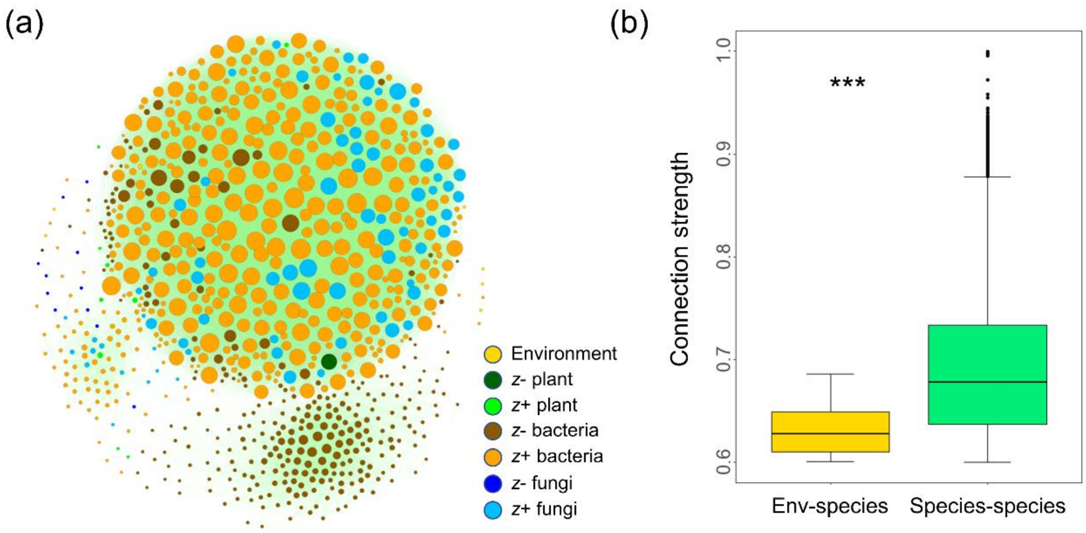

Figure S8. Correlation network analyses of soil environmental variables and indicator species of plant and soil microbial communities for restoration duration. Correlation network including soil environmental variables and *z*- and *z*+ indicator species of plant and soil microbial communities (a). Soil environmental variables and indicator species groups are indicated by node colors. The size of each node is proportional to its connectivity, which is the sum of the connection strengths ($|\rho|$) of each node with other connected nodes. A connection stands for a robust correlation ($|\rho| > 0.6$ and FDR-corrected $P < 0.001$). Positive and negative connections are indicated by green and gray edges, respectively. Boxplots of the absolute values of Spearman's correlation among soil environmental variables and the relative abundance of species and among the relative abundance of species from this network (b). Significant differences between boxes are determined using the non-parametric Mann–Whitney *U* test. Significant *P* value is represented by *** when $P < 0.001$.

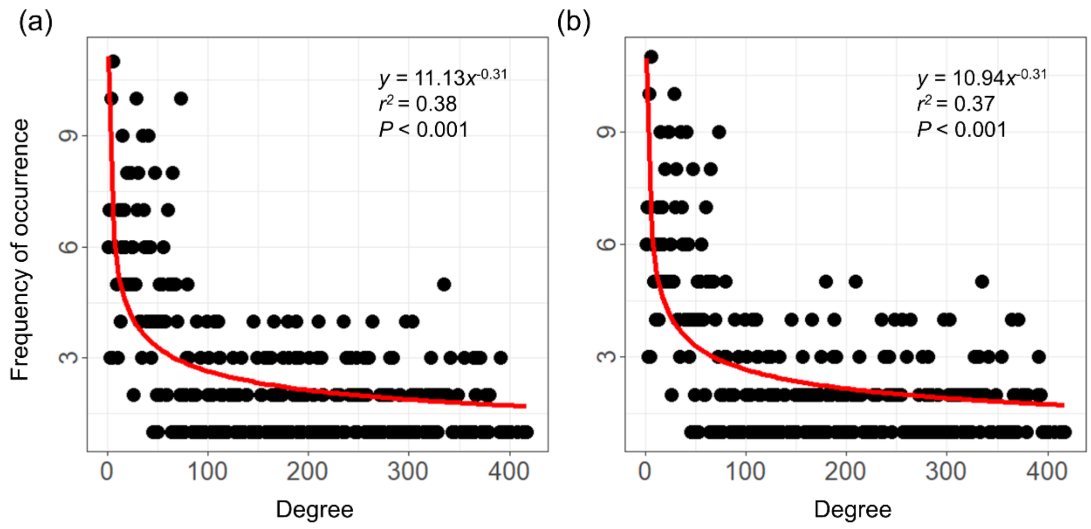

Figure S9. Degree distributions of nodes in correlation networks. Nodes are soil environmental variables and indicator species from the network in Figure S8a (a). Nodes are indicator species from the network in Figure 5a (b). Nodes show significant power-law distributions of edges, indicating non-random co-occurrence patterns for two correlation networks.

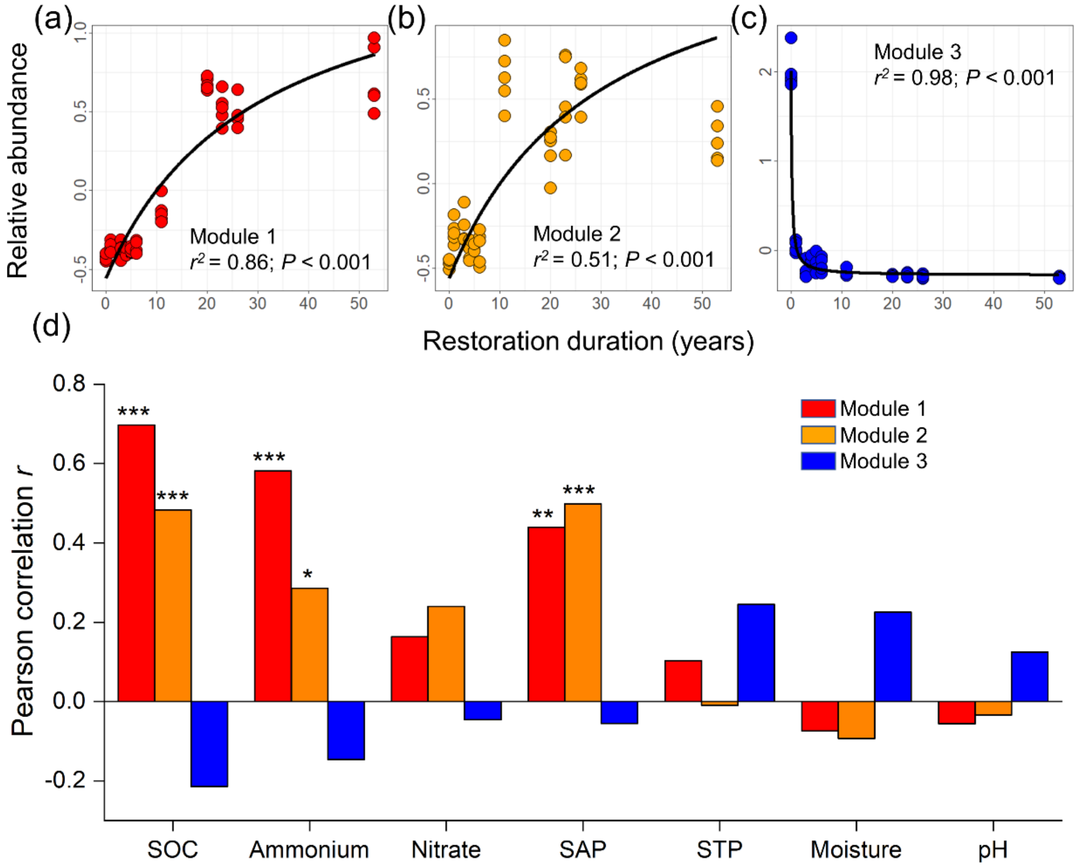

Figure S10. Relationships between SCB restoration duration and soil environmental variables and the relative abundance of the three main modules in the network. Relationships between SCB restoration duration and the relative abundance of module 1 (a), module 2 (b) and module 3 (c) in the network. Pearson correlations between soil environmental variables and the relative abundance of three main modules (d). Significant P values are represented by *** when $P < 0.001$, ** when $P < 0.01$ and * when $P < 0.05$.

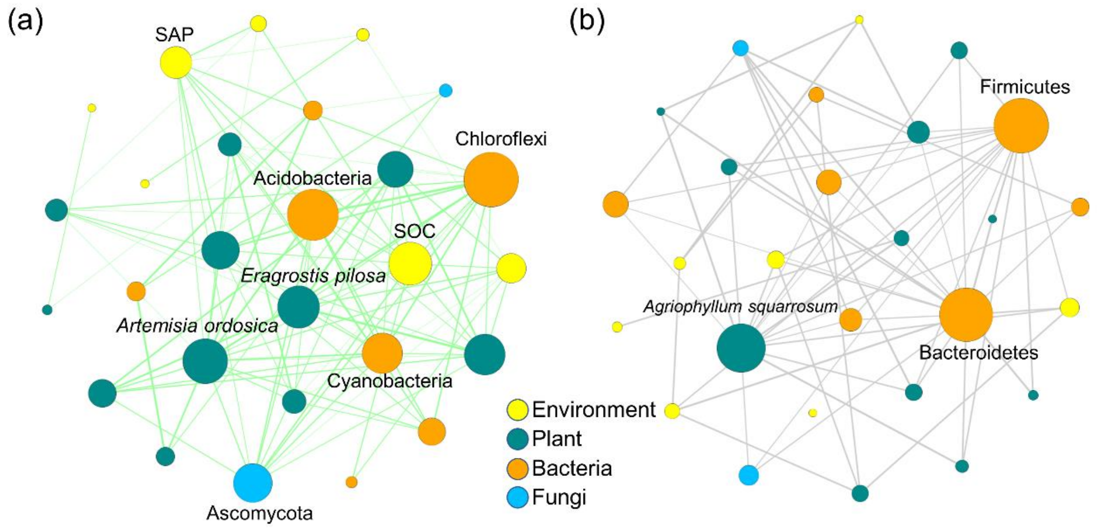

Figure S11. Correlation network including indicator species and soil environmental variables when soil indicator species were at the phylum level. Networks constructed based on positive (ρ > 0.6; a) and negative (ρ < -0.6; b) correlations. Indicator species and environmental variables are indicated by node colors. The size of each node is proportional to its connectivity, which is the sum of the connection strengths (|ρ|) of each node with other connected nodes. Nodes with greater connectivity are labeled with plant species, soil phyla, or environmental variables.

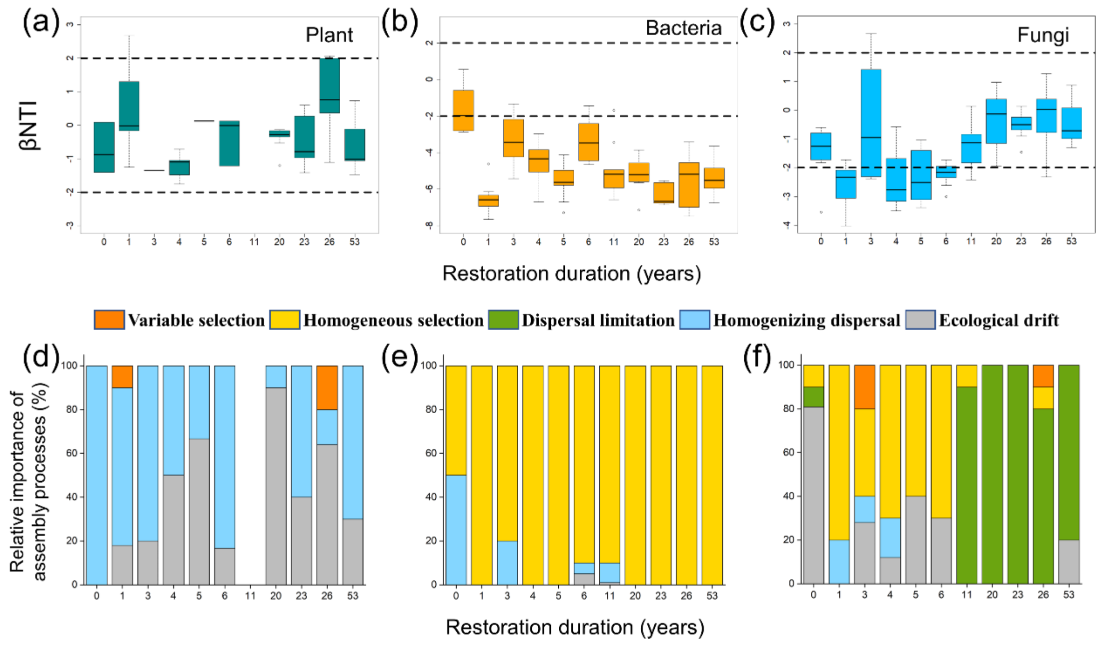

Figure S12. Temporal change in βNTI distributions and the relative importance of ecological processes mediating community assembly. Patterns of βNTI along restoration duration for plants (a), soil bacteria (b) and fungi (c). Horizontal dashed lines indicate upper and lower significance thresholds at βNTI = +2 and -2, respectively. The relative importance of ecological processes mediating community assembly of plants (d), soil bacteria (e) and fungi (f) with restoration duration. The plant community that has been restored for 11 years consists of only one species, thus it is not possible to assess the importance of ecological processes that assembled this community.

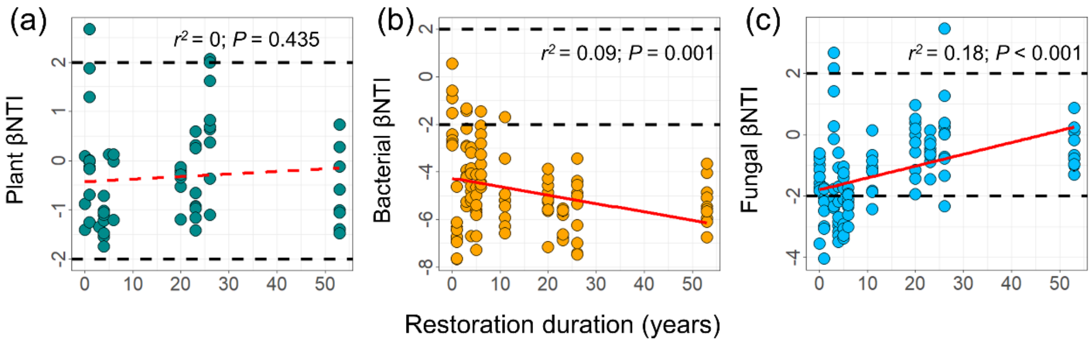

Figure S13. Relationships between βNTI and SCB restoration duration. Relationships between βNTI and SCB restoration duration for plant (a), soil bacterial (b) and fungal (c) communities. Horizontal dashed lines indicate upper and lower significance thresholds at βNTI = +2 and -2, respectively. The red fitted lines are from linear regression. The solid and dotted lines represent statistically significant ($P \leq 0.05$) and nonsignificant ($P > 0.05$) relationships, respectively.

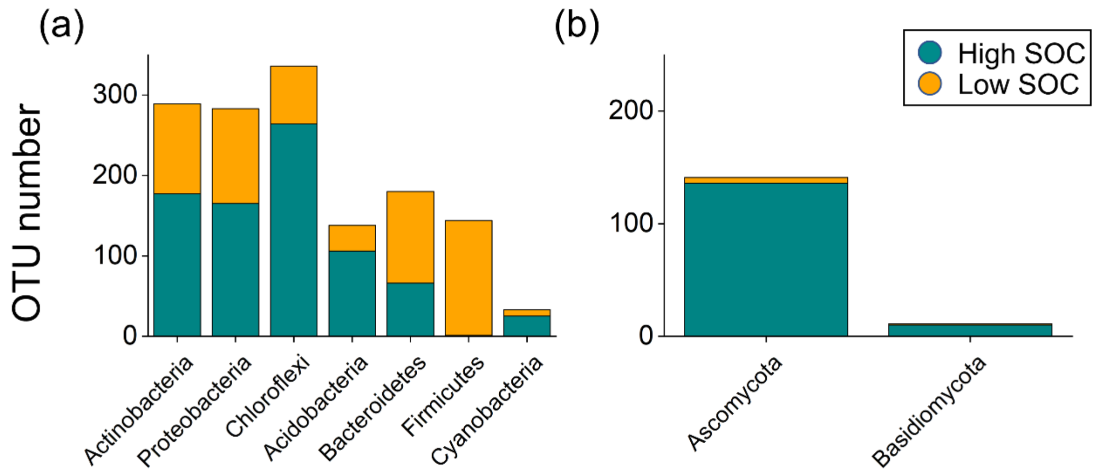

Figure S14. Number of indicator OTUs at the phylum level for SOC in two ecological clusters (high SOC and low SOC). Number of indicator bacterial (a) and fungal (b) OTUs at the phylum level for SOC in two ecological clusters (high SOC and low SOC). Only the dominant phyla are shown.

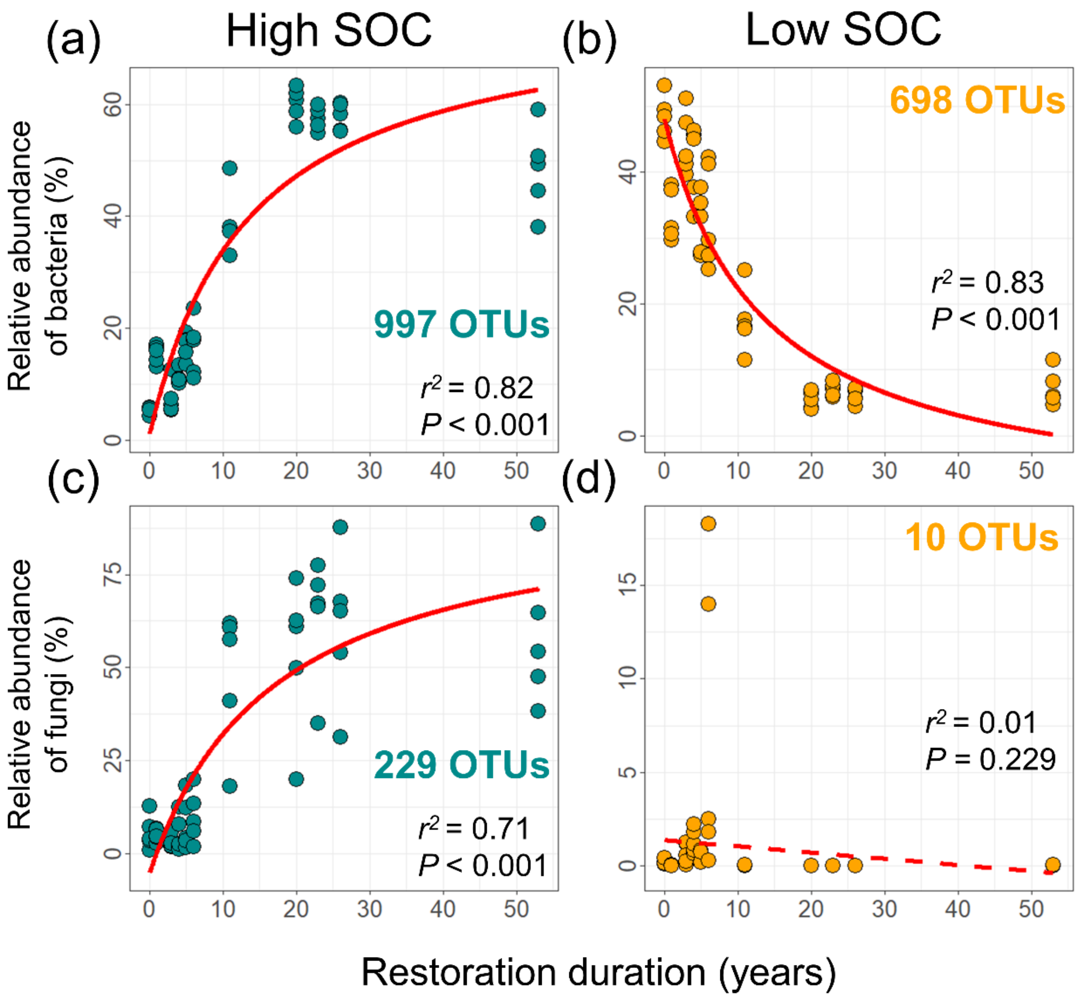

Figure S15. Relationships between restoration duration and the relative abundance of ecological clusters (high SOC and low SOC). The *z*+ and *z*- indicator bacterial and fungal OTUs for SOC were assigned to two ecological clusters (high SOC and low SOC), respectively. Relationships between restoration duration and the relative abundance of *z*+ and *z*- bacteria (a, b) and fungi (c, d) for SOC. The red fitted lines are from linear and nonlinear regression. The solid and dotted lines represent statistically significant ($P \leq 0.05$) and nonsignificant ($P > 0.05$) relationships, respectively.

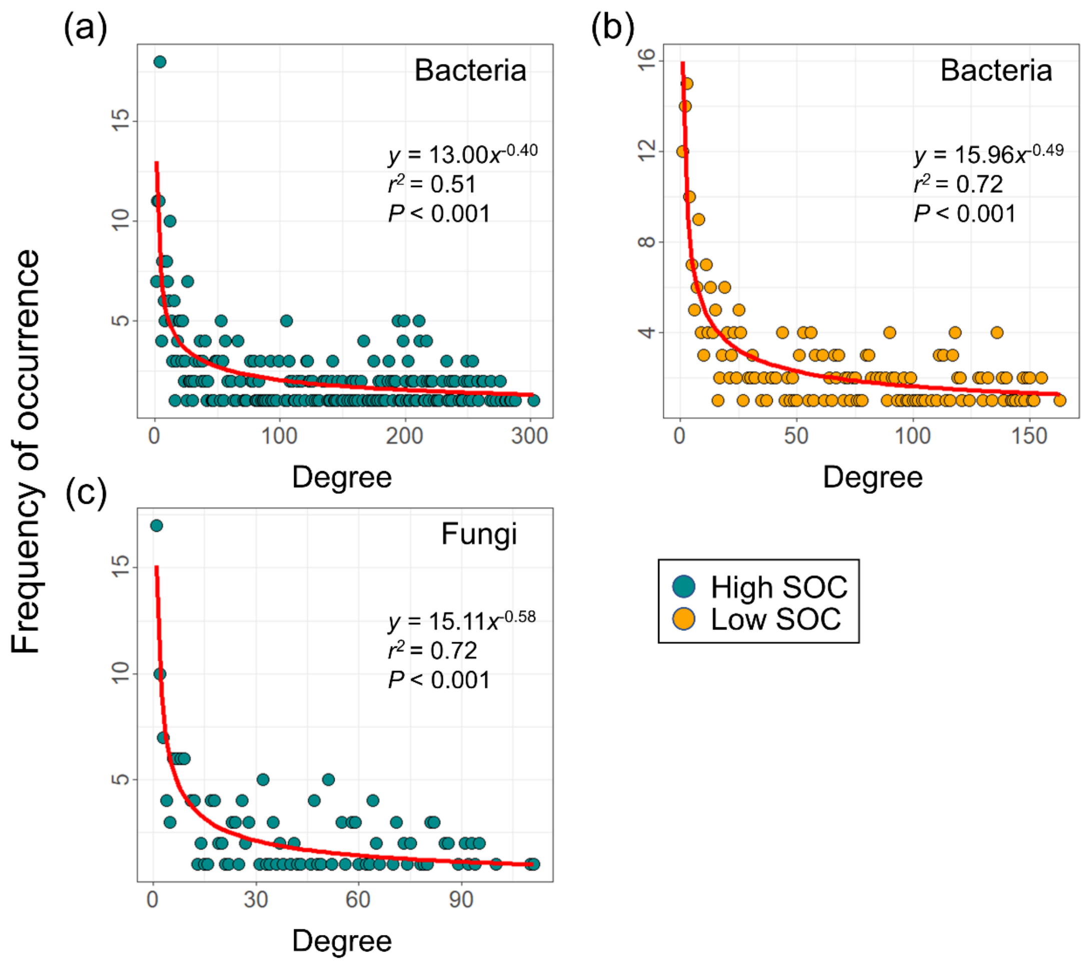

Figure S16. Degree distributions of the two ecological clusters of soil bacterial and fungal OTUs sharing ecological preference for SOC in co-occurrence networks. Nodes of each ecological cluster of soil bacterial OTUs show a significant power-law distribution of edges (a, b), indicating that OTUs tend to co-occur with others within the same cluster. Nodes of ecological cluster of soil fungal OTUs sharing ecological preference for high SOC show a significant power-law distribution of edges (c), while only 10 nodes in ecological cluster with a preference for low SOC showed no such distribution (not shown in the figure).

Table S1. ANOSIM of the composition of plant and soil microbial communities between restored and natural ecosystems.

Data set	Restoration duration (years)										
	0	1	3	4	5	6	11	20	23	26	53
Plant	1.00***	1.00***	1.00***	0.98***	0.87***	0.90***	0.67***	1.00***	0.78***	0.95***	0.89***
Bacteria	1.00***	1.00***	1.00***	1.00***	1.00***	1.00***	0.74***	0.50***	0.48***	0.44***	0.80***
Fungi	0.92***	0.94***	0.94***	0.99***	0.92***	0.97***	0.51***	0.21	0.13	0.41**	0.78**

Significant *P* values are represented by *** when $P < 0.001$, ** when $P < 0.01$ and * when $P < 0.05$.

Table S2. Indicator plant species responding to SCB restoration duration and their thresholds.

Plant species	Class	Family	Genus	*Z* score	Threshold (year)
Negative (*z*-) indicator species					
Agriophyllum squarrosum	Dicotyledoneae	Chenopodiaceae	*Agriophyllum*	13.35	8.5
Corispermum patelliforme	Dicotyledoneae	Chenopodiaceae	*Corispermum*	3.90	4.5
Positive (*z*+) indicator species					
Euphorbia humifusa	Dicotyledoneae	Euphorbiaceae	*Euphorbia*	13.14	39.5
Artemisia ordosica	Dicotyledoneae	Compositae	*Artemisia*	11.09	3.0
Eragrostis pilosa	Monocotyledoneae	Poaceae	*Eragrostis*	9.51	15.5
Psammochloa villosa	Monocotyledoneae	Poaceae	*Psammochloa*	8.98	20.0
Stipa glareosa	Monocotyledoneae	Poaceae	*Stipa*	8.75	24.5
Setaria viridis	Monocotyledoneae	Poaceae	*Setaria*	8.73	39.5
Tugarinovia mongolica	Dicotyledoneae	Compositae	*Tugarinovia*	8.02	24.5
Cirsium setosum	Dicotyledoneae	Compositae	*Cirsium*	7.48	15.5
Corispermum mongolicum	Dicotyledoneae	Chenopodiaceae	*Corispermum*	4.57	20.0

Table S3. Number of indicator species and threshold values of plant and soil microbial communities in response to soil environmental variables by TITAN.

Soil environmental variable		Number of indicators		Threshold	
		z-	*z+*	*z-*	*z+*
SOC ($mg\ kg^{-1}$)	Plant	2	10	2.81	2.87
	Bacteria	698	997	2.83	2.84
	Fungi	10	229	2.68	2.94
Ammonium ($mg\ kg^{-1}$)	Plant	2	7	0.09	0.40
	Bacteria	437	535	0.01	0.40
	Fungi	9	64	-0.11	0.32
Nitrate ($mg\ kg^{-1}$)	Plant	-	-	-	-
	Bacteria	48	67	2.19	3.19
	Fungi	2	26	2.19	3.25
SAP ($mg\ kg^{-1}$)	Plant	2	5	1.37	2.30
	Bacteria	279	782	1.25	2.68
	Fungi	7	231	1.25	3.09
STP ($mg\ kg^{-1}$)	Plant	2	5	89.55	136.18
	Bacteria	194	198	78.21	108.95
	Fungi	11	41	78.21	108.95
Moisture (%)	Plant	3	3	0.61	1.29
	Bacteria	102	127	0.61	1.29
	Fungi	36	7	0.61	1.29
pH	Plant	-	-	-	-
	Bacteria	53	213	8.73	8.96
	Fungi	24	4	8.73	8.89

Table S4. Pearson correlations between the relative importance of stochasticity in controlling soil microbial community succession and soil environmental variables.

Stochasticity	SOC	Ammonium	Nitrate	SAP	STP	Moisture	pH
Soil bacteria	-0.33*	-0.13	-0.03	-0.15	0.01	0.09	0.12
Soil fungi	0.47***	0.41**	0.14	0.41**	0.06	-0.17	0.11

Significant *P* values are represented by *** when $P < 0.001$, ** when $P < 0.01$ and * when $P < 0.05$.
